# G-quadruplex binders as cytostatic modulators of innate immune genes in cancer cells

**DOI:** 10.1093/nar/gkab500

**Published:** 2021-06-17

**Authors:** Giulia Miglietta, Marco Russo, Renée C Duardo, Giovanni Capranico

**Affiliations:** Department of Pharmacy and Biotechnology, Alma Mater Studiorum—University of Bologna, via Selmi 3, 40126 Bologna, Italy; Department of Pharmacy and Biotechnology, Alma Mater Studiorum—University of Bologna, via Selmi 3, 40126 Bologna, Italy; Department of Pharmacy and Biotechnology, Alma Mater Studiorum—University of Bologna, via Selmi 3, 40126 Bologna, Italy; Department of Pharmacy and Biotechnology, Alma Mater Studiorum—University of Bologna, via Selmi 3, 40126 Bologna, Italy

## Abstract

G-quadruplexes (G4s) are non-canonical nucleic acid structures involved in fundamental biological processes. As G4s are promising anticancer targets, in past decades the search for effective anticancer G4 binders aimed at the discovery of more cytotoxic ligands interfering with specific G4 structures at oncogenes or telomeres. Here, we have instead observed a significant activation of innate immune genes by two unrelated ligands at non-cytotoxic concentrations. The studied G4 binders (pyridostatin and PhenDC3) can induce an increase of micronuclei triggering the activation of the cytoplasmic STING (stimulator of interferon response cGAMP interactor 1) signaling pathway in human and murine cancer cells. Ligand activity can then lead to type I interferon production and innate immune gene activation. Moreover, specific gene expression patterns mediated by a G4 binder in cancer cells correlate with immunological hot features and better survival in human TCGA (The Cancer Genome Atlas) breast tumors. The findings open to the development of cytostatic G4 binders as effective immunomodulators for combination immunotherapies in unresponsive tumors.

## INTRODUCTION

G-quadruplexes (G4s) are non-canonical DNA secondary structures constituted by stacked guanine quartets forming a planar system held by Hoogsteen hydrogen bonds (1–3). Genome localization of G4 structures is not random, but rather they are typically enriched in control regions, such as active gene promoters, telomeres and replication origins, suggesting regulatory roles during basic genome functions (2–4). However, G4 structures can interfere with replication and transcription processes leading to double-strand DNA breaks (DSBs) and genome instability (5). Hundreds of small molecules are known to bind specifically to G4 structures as compared with a DNA duplex (6). Although these G4 binders are extensively investigated to discover cytotoxic analogs as new effective anticancer drugs (1,7–9), only few of them have entered clinical trials up to now (8,10).

G4 binders can stabilize nuclear G4 structures inducing DSBs in cancer cells (10–13), and recent evidence shows that the mechanism can involve R-loops (5,12,13). R-loops are another non-canonical DNA secondary structure wherein a duplex DNA is melted and one strand is annealed to an RNA strand. R-loops can form on highly transcribed genes (14–16) and can lead to replication stress, DSBs and genome instability (17–19). We recently showed that three structurally unrelated G4 binders increased nuclear R-loops due to the spreading of pre-existing structures at transcribed genes (5,12). Interestingly, G4 binders [including pyridostatin (PDS), a well-known ligand (8)] could also trigger the formation of micronuclei in an R-loop-dependent manner in human osteosarcoma U2OS cells at non-cytotoxic concentrations (12). Micronuclei can be a source of cytoplasmic DNAs that can be recognized by DNA sensors, such as cGAS (cyclic GMP–AMP synthase), which can activate STING (stimulator of interferon response cGAMP interactor 1, alias TMEM173) and the expression of type I interferon (IFN) genes and IFN-stimulated genes (ISGs) (20–22). Although recent advances clearly point to the potential of harnessing the innate immune system for cancer immunotherapy (23), whether G4 binders can induce immune genes in cancer cells has not yet been determined.

Here, we focus on immune gene response to G4 binders at non-cytotoxic concentrations and define the mechanism of gene activation. In addition, we show that these ligands can act with a genome-wide effect by enhancing micronuclei formation leading to activation of innate immune genes in cancer cells. Our findings therefore open to the development of G4 binders as anticancer modulators of the immune system for new immunotherapeutic combinations.

## MATERIALS AND METHODS

### Cell lines and compounds

Human MCF-7 (breast cancer) and MRC5 (derived from a normal lung tissue) cells were obtained from American Type Culture Collection. Murine wild-type (wt) and STING gene-CRISPR knockout (KO) melanoma B16-F10 cell lines were kindly provided by R. Greenberg (20). B16, MRC5 and MCF-7 cell lines were grown as monolayers in Dulbecco’s modified Eagle medium or RPMI 1640 medium respectively, supplemented with 10% fetal bovine serum (FBS) (Gibco) and 1% l-glutamine (Gibco) in a humidified incubator at 37°C and 5% CO_2_. Cell lines were routinely tested for mycoplasma contamination (Sigma-Aldrich) and identity by STR (BMR Genomics).

PDS (Selleckchem) was dissolved in water at 5 mM. H151 (TOCRIS, Biotechne) and PhenDC3 (Merck) were dissolved in dimethyl sulfoxide (DMSO) at 3 and 10 mM, respectively. All compounds were stored at −20°C and diluted immediately prior to use.

### Cell growth, MTT assay and cell transfection

For MCF-7 cell growth, cells (3 × 10^5^) were seeded in six-well plates and treated the next day with PDS (10 μM). Cells were counted every day and reported as cells/cm^2^. For MTT assays, MCF-7 cells (5 × 10^4^) were seeded and, after 24 h, treated with the compounds at several different concentrations for 24 h. After drug removal, cells were cultured for 48 h in drug-free medium. Then, thiazolyl blue tetrazolium bromide (MTT) solution was added to each well and incubated for 1 h at 37°C. Next, the medium was removed and 300 μl DMSO was added and incubated for 1 h at room temperature (RT). Then, absorbance at 595 nm was measured using a multiplate reader. Linear regression parameters were determined to calculate IC_50_ (concentration inhibiting cell growth by 50%) values (GraphPad Prism 8). MCF-7 cells were transfected with pre-designed siRNAs (against STING: ID#128591, siRNA1, and ID#128592, siRNA2; Ambion) using Lipofectamine RNAImax (Invitrogen) for 72 h following manufacturer’s instructions. Salmon sperm DNA (2 μg/ml) was transfected using Lipofectamine 2000 (Invitrogen) for 24 h.

### RNA extraction and RT-qPCR

Treated and untreated cells were washed with phosphate-buffered saline (PBS) and centrifuged. Cell pellets were resuspended in 0.8 ml of TRI Reagent™ (#AM9738, Invitrogen), vortexed and incubated for 5–10 min at RT. Then, samples were added with 0.16 ml of chloroform (Fisher Scientific), vortexed, incubated for 5 min at RT and centrifuged at 4°C for 15 min at 20 000 × *g*. The upper phase was collected and nucleic acids were isopropanol precipitated. Samples were digested with DNase I, phenol extracted and then RNA was ethanol precipitated. RNA quality was checked with agarose gels, and 1 μg of RNA was used to prepare cDNA with SuperScript III (Invitrogen) according to manufacturer’s instructions using random (N6) and poly(T) primers. After primer annealing, retrotranscription reaction was performed for 50 min at 50°C. Then, RNA was alkaline hydrolyzed and ethanol precipitated. Quantitative PCR (RT-qPCR) was performed using 500 nM of specific primers in SsoAdvanced Universal SYBR Green Supermix (Bio-Rad) with Applied Biosystems StepOne thermocycler. Quantification and analyses were performed using StepOne Software v2.2.3. Specificity of PCR products was routinely checked with melting curves and agarose gel electrophoresis. PCR primers are reported in Supplementary Table S2.

### Illumina RNA sequencing and data analyses

MCF-7 cells (1.0 × 10^6^) were seeded in 6 mm dishes, treated with PDS (10 μM) for 24 h and then cultured in drug-free medium for further 72 h. Total RNA was extracted by using the NucleoSpin RNA kit (#740955.50, Macherey-Nagel) or TRI Reagent™ (#AM9738, Invitrogen) following manufacturer’s instructions. RNA samples were depleted of ribosomal RNAs with Ribo-Zero rRNA Removal Kit (Illumina) and libraries prepared with NEBNext Ultra Directional RNA Library Prep Kit for Illumina (NEB, #E7420S) following manufacturer’s instructions. RNA sequencing was performed on Illumina HiSeq4000 platform (pair-end 2 × 150 bp) at Biodiversa (Rovereto, TN, Italy). RNA-seq reads were quality checked with FastQC v0.11.9 and trimmed with Trimmomatic v0.32 software (24). Libraries were then aligned to human genome (hg19) with HISAT2 program (25), and read abundance over transcripts was computed with StringTie v2.0 (26) tool using Ensembl GRCh37.87 gene reference. StringTie v2.0 transcript estimations were imported in R v4.0.0 and converted to read count using Bioconductor tximport v1.16.1 package (27). Read counts were batch corrected using the R package limma v3.44.3 library. Differential expression and PCA analyses were performed using Bioconductor DESeq2 v1.28.1 package (28) with default settings. Differentially expressed (DE) genes were selected by adjusted *P*-value <0.05. Gene set enrichment analysis (GSEA) was performed using Bioconductor fgsea v1.14.0 package (27) and MSigDB gene set database (version 7.0) with default settings. GSEA used as input DE gene tables obtained from DESeq2. Comparison of GSEA results and data plotting were performed using the Bioconductor tidverse v1.3.0 and enrichplot v1.8.1 packages (27), respectively. Gene sets of IRF, STAT and NF-κB targets were determined by using TRANSFAC database (29).

### Immunofluorescence microscopy

Fluorescence signals were determined following different procedures using cells in a coverslip. For STING, cells were fixed with 4% of formaldehyde for 10–15 min at RT and then incubated for 1 h at RT with 1% of bovine serum albumin (BSA), 10% FBS, 0.1% glycine and 0.1% Tween 20 under gentle shaking. Then, cells were stained with 0.5 μg/μl anti-STING antibody (#ab92605, Abcam) overnight at 4°C. For cGAS, cells were permeabilized with 0.5% Triton X-100 in PBS for 15 min at RT and then incubated with 1% BSA in PBS for 30 min at RT. Cells were then incubated with anti-cGAS antibody (clone D1D3G, #15102, Cell Signaling) for 1 h at RT. For pIRF3, cells were incubated in cold methanol for 10 min at −20°C. After a wash in PBS, cells were incubated with 5% of FBS and 0.3% of Triton X-100 in PBS for 60 min at RT under gentle shaking. Then, cells were incubated with an anti-pIRF3 (specific for phosphorylated Ser396 IRF3) (clone D6O1M, #29047, Cell Signaling) overnight at 4°C. For IRF3, cells were fixed with methanol chilled at −20°C for 5 min at RT. Permeabilization was performed with 0.1% Triton X-100 for 10 min at RT under gentle shaking. Next, cells were incubated with 1% BSA, 23 mg/ml glycine and 0.1% Tween 20 for 30 min at RT. Cells were then incubated with an anti-IRF3 antibody (clone 3H32L10, #703682, Thermo Fisher) overnight at 4°C. For S139-phosphorylated histone H2AX (γH2AX), cells were fixed with 4% of formaldehyde for 10 min at RT, permeabilized by 0.5% Triton X-100 in PBS for 15 min at RT and then incubated with 8% BSA in PBS for 30 min at RT. An anti-γH2AX antibody (#05-636, Millipore) was then added in 5% BSA for 1 h at RT. For all factors, cells were finally incubated with a secondary antibody and stained with DAPI (3.3 μg/ml) (Merck) for 20 min. Secondary antibodies were Alexa Fluor 488 anti-rabbit IgG (#A11008, Life Technologies) and Alexa Fluor 488/594 anti-mouse IgG (#A11011, #A11037 Life Technologies). Micronuclei were identified by DAPI staining: cells were fixed with 4% paraformaldehyde for 15 min, permeabilized with 0.5% Triton X-100 for 15 min at RT, washed three times in PBS and incubated with 3.3 μg/ml DAPI for 20 min. G4 foci were detected using BG4 (kindly provided by S. Balasubramanian) as described previously (12). Slides were visualized at RT by using a fluorescence microscope (Eclipse TE 2000-S, Nikon) equipped with an AxioCam MRm (Zeiss) digital camera. After fluorescence data acquisition, analyses were performed with ImageJ software. Graphs were prepared with GraphPad Prism 8.

### Protein extraction and western blotting

Whole cell protein extracts were prepared by lysing cells in 4% sodium dodecyl sulfate (SDS), 20% glycerol and 125 mM Tris–HCl, pH 6.8, and protease and phosphatase inhibitor cocktails (Thermo Fisher). For cell fractionation, cells were scraped, resuspended in an isotonic buffer (10 mM HEPES, 200 mM mannitol, 70 mM sucrose, 1 mM EDTA, pH 7.6), added with protease and phosphatase inhibitors, and incubated for 30 min on ice. Then, cells were gently lysed with a Potter homogenizer, and nuclei were pelleted by centrifugation at 800 × *g* for 10 min at 4°C and washed with an isotonic buffer. The supernatant cytoplasmic fraction was centrifuged for 10 min at 800 × *g* to remove debris. Nuclei were resuspended in 20 mM HEPES, pH 7.9, 10 mM NaCl, 3 mM MgCl_2_, 0.1% NP-40, 10% glycerol, 0.2 mM EDTA and 1 mM DTT and incubated on ice for 10 min. Next, pelleted nuclei were washed with 20 mM HEPES, pH 7.9, 0.2 mM EDTA, 20% glycerol and 1 mM DTT and resuspended in 20 mM HEPES, pH 7.9, 400 mM NaCl, 0.2 mM EDTA, 1 mM DTT, 20% glycerol and 2% SDS. Protein samples were sonicated for 5 min with a Bioruptor sonifier (Diagenode), quantified with the Lowry method and stored at −80°C until use. Proteins (20–30 μg) were electrophoresed on pre-cast gels (Invitrogen) and transferred to a nitrocellulose membrane in 25 mM Tris–HCl, pH 7.6, 192 mM glycine and 20% methanol at 70 V for 2 h. Membranes were incubated for 1 h in 5% non-fat milk, 0.5% Tween 20 TBS solution under gentle shaking at RT and then with primary antibodies overnight at 4°C. Primary antibodies were STING (dilution 1/1000) (#ab92605 and #ab181125, Abcam), pIRF3 (Ser385) (dilution 1/200) (#PA5-36775, Invitrogen), IRF3 (dilution 1/500) (clone 3H32L10, #703682, Thermo Fisher), topoisomerase I (C15) (dilution 1/100) (#sc5342, Santa Cruz) and GAPDH (dilution 1/20 000) (#sc32233, Santa Cruz). Then, membranes were incubated 90 min at RT with horseradish peroxidase-linked secondary antibodies. They were anti-mouse IgG (1/1000 dilution) (#sc2005, Santa Cruz), anti-rabbit IgG (1/10 000 dilution) (#ab205718, Abcam) and anti-goat IgG (1/2000) (#sc2922, Santa Cruz). All western blots analyses were performed with three biological replicates, and band intensity values were reported as means ± SEM.

### Determination of cellular cGAMP and secreted cytokines

Levels of the dinucleotide 2′,3′-cyclic GAMP (cGAMP) were measured in whole cell extracts. Cell pellets were resuspended in RIPA buffer [20 mM Tris–HCl, pH 7.5, 150 mM NaCl, 1 mM EDTA, 1 mM EGTA, 1% NP-40, 2 mM DTT, 0.5 mM phenylmethylsulfonyl fluoride (PMSF) and Halt Protease Inhibitor Cocktail], incubated for 30 min on ice and centrifugated for 20 min at 12 000 × *g* at 4°C. Pellets were then discarded and supernatants were used to determine cGAMP levels with the Direct 2′,3′-Cyclic GAMP Enzyme Immunoassay Kit (#K067-H1, Arbor Assays).

Secreted cytokines were measured in cell medium supernatants. Culture medium of untreated and PDS (10 μM)-treated cells was collected and protease inhibitors (1 μg/ml pepsatin, leupeptin and aprotinin, 2 mM DTT and 0.5 mM PMSF) were then added. Then media were concentrated around 30-fold by using Pierce Protein Concentrator PES, 3k MWCO, 5–20 ml (#88525, Thermo Fisher). IFNB protein levels were quantified with a Human IFN-beta Quantikine ELISA Kit (#DIFNB0, R&D Systems) following manufacturer’s instructions. To detect cytokines and chemokines in concentrated cell culture medium, a Human Cytokine Array Kit (#ARY005B, R&D Systems) was used following manufacturer’s instructions.

### TCGA breast tumor analyses

FPKM-UQ-normalized gene expression data of the breast cancer (BRCA) patient cohort (*n* = 1217) of the public GDC The Cancer Genome Atlas (TCGA) database were collected from Xena Functional Genomics Explorer (https://xenabrowser.net). Only primary tumors (*n* = 1064) were used in subsequent analyses. Data regarding tumor-infiltrating immune cells of TCGA BRCA samples were from supplementary data of the study (30).

Using a gene list of PDS upregulated genes (adjusted *P*-value <0.05) computed in the PDS (day 4) versus CT (day 4) contrast with Bioconductor DESeq2 package (28), a *k*-means cluster analysis of primary BRCA data was performed with CRAN package Hmisc v4.4 and Bioconductor ComplexHeatmap v2.4.3 R libraries (27). Main transcription factors regulating a given gene set were determined with iRegulon v1.3 tools (31) on Cytoscape v3.8.2 platform (32) using default settings. GSEA enrichment score was determined with ssGSEA function from GSVA R library. KEGG pathway gene sets were from MSigDB v6.2. Immune cell presence in tumor samples was computed with CIBERSORT (33). Spearman correlation coefficient of ssGSEA enrichment scores, immunological tumor features and KEGG pathways were determined using Bioconductor corrplot v0.84 R library (27). Survival analysis and plotting were performed using Bioconductor survminer v0.4.8 and ggplot2 v3.3.2 R libraries (27). Survival analysis was performed for the top and bottom 5% of enrichment score-ranked samples of TCGA BRCA primary tumors.

### Statistics

All results were from at least three biological replicates. Statistical significance was determined with a multiple *t*-test (without corrections for multiple comparisons) between pairs of conditions for micronuclei and cGAS-positive micronuclei counts, gene expression analysis, cGAMP and interferon production. The chi-square test was used for pIRF3-positive cell analyses between pairs of conditions. The Kolmogorov–Smirnov test was used for G4 and γH2AX fluorescence quantification between pairs of conditions. Significant differences between the indicated pairs of conditions are shown by asterisks (*P-value <0.05; **P-value <0.01; ***P-value <0.001; ^****^P-value <0.0001). Differential gene expression analysis was performed using Welch’s *t*-test. Significance of GSEA was assessed by comparing each gene set enrichment score with the set of scores computed with randomly assigned phenotypes (*n* = 1000). Correlation analysis of TCGA BRCA expression data was tested using the Spearman correlation test with the cor.test function (R library stats v3.6.2). Comparison of survival curves was tested with the log-rank test using R package survminer v0.4.8.

## RESULTS

### Non-cytotoxic PDS concentrations induce micronuclei and immune genes in MCF-7 cancer cells

**Figure 1.**
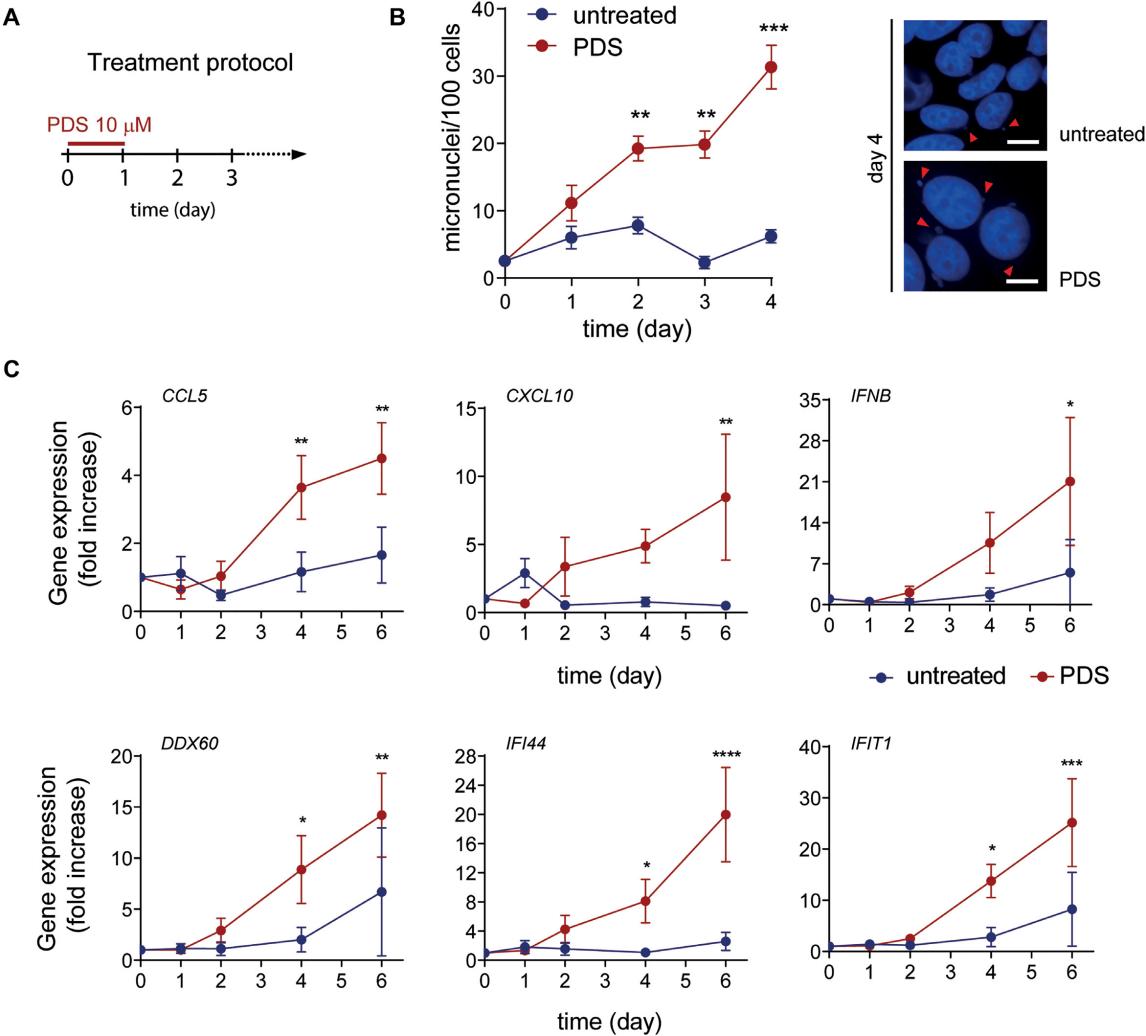
PDS-induced micronuclei accumulation precedes immune gene activation. (**A**) Experimental scheme. (**B**) Kinetics of micronuclei accumulation in PDS (10 μM)-treated MCF-7 breast cancer cells (left) and representative images of micronuclei in untreated and PDS-treated cells at day 4 (right). Data show means ± SEM of at least three biological replicates. Scale bar is 10 μm. (**C**) Expression fold increase of IRF3-induced genes (*CCL5*, *CXCL10* and *IFNB*) and ISGs (*DDX60*, *IFIT1* and *IFI44*) in MCF-7 cells treated with PDS (10 μM) as measured by RT-qPCR. Data show means ± SEM of at least three biological replicates. In all graphs, significance was calculated with a multiple unpaired *t*-test: *P-value <0.05; **P-value < 0.01; ***P-value <0.001; ^****^P-value <0.0001.

We set out to establish whether PDS can activate an immune gene response in human MCF-7 breast cancer cells at non-cytotoxic concentrations. Therefore, we first measured the cytotoxic potency of PDS, showing that its IC_50_ (concentration inhibiting 50% of cell survival) for 24-h treatments is 81.6 μM in MCF-7 cells (Supplementary Figure S1A). At a lower dose, 10 μM, PDS only slightly affected MCF-7 cell growth (Supplementary Figure S1B) even though it increased the levels of G4 and γH2AX (phosphorylated S139 H2AX histone) foci (Supplementary Figure S2). Thus, a non-cytotoxic concentration of PDS can stabilize nuclear G4s likely inducing DSBs in MCF-7 cells, in agreement with findings in other cell models (11,12). Next, to determine the kinetics of micronuclei accumulation in MCF-7 cells, we set out an experimental scheme in which cells were treated with 10 μM PDS for 24 h and then let recover in drug-free medium for different time periods (Figure 1A). Micronuclei accumulated over time in PDS-treated cells with a maximum (15-fold change) at day 4 (Figure 1B). A slight micronuclei increase (2–3-fold) was also observed in untreated cells (Figure 1B). As the doubling time of MCF-7 is around 40 h (Supplementary Figure S1B), the results are consistent with micronuclei forming at subsequent mitoses following PDS treatment (20,21). Expression kinetics of selected of IRF3 (*IFNB*, *CCL5* and *CXCL10)* (34) and IFNB (*IFIT1*, *DDX60* and *IFI44*) (35) target genes showed that these innate immune genes were effectively activated by PDS at later times (days 4 and 6), with a slight increase at day 2 for some of them (Figure 1C). We also noted that the tested genes were increased, but to a lower extent, in untreated cells, which may parallel the slight induction of micronuclei (Figure 1B and C). Thus, innate immune genes were activated by non-cytotoxic PDS concentrations following an earlier micronuclei induction.

### Inflammatory immune gene pathways are activated by PDS at a non-cytotoxic concentration

As the above data showed that immune genes were fully activated at day 4, we hence determined PDS-induced changes of gene expression profiles at that time point by using RNA-seq Illumina technology. We compared PDS-treated cells at day 4 versus untreated cells at day 4 versus untreated cells at day 0 (Figure 1A) using four biological replicates of each sample. Quality checks of batch-corrected and normalized sequence data established that replicates clustered close together in three distinct groups (Supplementary Figure S3), showing consistent overall read distributions within each group.

**Figure 2.**
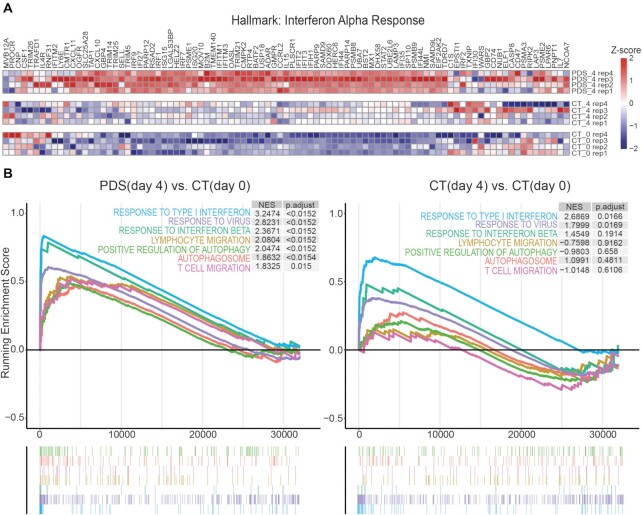
PDS induces a marked type I interferon response in MCF-7 breast cancer cells. (**A**) Heatmap showing *Z*-scores (color coded) for each RNA-seq sample (row) of genes (columns) belonging to MSigDB hallmark ‘Interferon Alpha Response’ gene set. (**B**) GSEA plot of upregulated GO biological processes for the PDS (day 4) versus CT (day 0) and CT (day 4) versus CT (day 0) contrasts. Top: enrichment score line plot. Bottom: position of genes along the ranked list of genes. Normalized enrichment score (NES) and adjusted *P*-values are indicated.

Comparison of gene read count data showed that gene expression profiles were changed at day 4 in both PDS-treated and untreated cells in comparison to untreated cells at day 0. However, the number of altered genes was higher in PDS-treated than in untreated cells (3444 and 1643 genes, respectively; Supplementary Figure S4A and B and Supplementary Data SD1 and SD2). In particular, inflammatory immune genes were increased by PDS and slightly induced in untreated cells as compared with day 0 (see, for instance, the ‘Interferon Alpha Response’ hallmark gene set, Figure 2A). GSEA of Gene Ontology (GO) biological processes showed that a subset of immune-related pathways, such as response to type I interferon, were activated somewhat in untreated cells, but prominently in PDS-treated cells (Figure 2B, Supplementary Figure S4C and D and Supplementary Data SD3 and SD4). Transcriptional response in untreated cells is consistent with the slight micronuclei increase observed at day 4 (Figure 1B). In addition, other specific GO biological processes, such as lymphocyte and T-cell migration, and autophagy-related terms were enriched in PDS-treated cells only (Figure 2B). A direct comparison of expression increase of genes belonging to MSigDB hallmark ‘Interferon Alpha and Beta Response’ gene set showed that these genes were increased at significantly higher levels in PDS-treated than untreated cells (Supplementary Figure S4C). Thus, PDS-dependent gene expression alterations were much larger than those observed in untreated cells.

**Figure 3.**
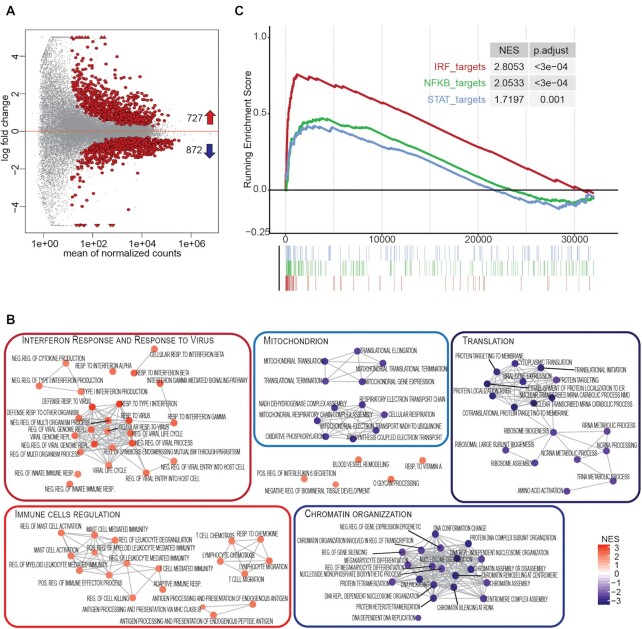
Specific effects of PDS on immune gene pathways. (**A**) MA plot of DE genes (red dots) in PDS (day 4) versus CT (day 4) contrast (*q*-value <0.05). Upward and downward arrows indicate the number of up- and downregulated genes, respectively. (**B**) Enrichment map showing GSEA results for PDS (day 4) versus CT (day 4) DE genes in GO ‘Biological Process’ gene sets (nodes) clustered by overlapping gene presence (edges). Gene sets are grouped into functional modules. Node color indicates NES as indicated on the right. Only the top 50 positively (red) and top 50 negatively (blue) enriched GO processes are shown. All nodes have adjusted *P*-values <0.005. (**C**) GSEA plot of enriched transcription factor target genes from TRASFAC database (29) for the PDS (day 4) versus CT (day 4) contrast. Top: enrichment score line plot. Bottom: position of genes along the ranked list of genes. NES and adjusted *P*-values are indicated.

A direct comparison of PDS-treated versus untreated cells at day 4 showed that 727 and 872 genes were up- and downregulated by PDS, respectively (Figure 3A, Supplementary Figure S5A and Supplementary Data SD5). The upregulated genes are markedly related with immune response pathways, including type I interferon responses, inflammatory antiviral responses and immune cell regulation networks (Figure 3B, Supplementary Figure S5B and Supplementary Data SD6–SD9). PDS-increased expression of six immune genes as detected with RT-qPCR (Figure 1C) was confirmed by RNA-seq data (Supplementary Table S1). At the same time, many pathways were reduced, including chromatin regulations, mitochondrial functions and translational processes (Figure 3B and Supplementary Figure S5A), which may be consistent with a slight delay of MCF-7 cell growth (Supplementary Figure S1B). Even though many cytokine genes were activated by PDS, we could not detect a general activation of SASP (senescence-associated secretory phenotype) (36), but rather its downregulation (Supplementary Figure S5C). Thus, RNA-seq data support that a non-cytotoxic PDS dose can induce a marked activation of type I interferon response genes, while slowing down basic metabolic processes in human cancer cells.

### PDS triggers the activation of the cGAS–STING–IRF3 pathway in cancer cells

To determine the mechanisms of immune gene activation by non-cytotoxic PDS, we next investigated enrichment rates of target genes of transcription factors in the PDS-altered gene set (Supplementary Figure S5A). Target genes of IRF1–7, STAT1–9 and NF-κB transcription factors (Supplementary Data S10D) were among the top 10 target gene sets upregulated by PDS, whereas those of ERF2 factors were among the most downregulated genes (Figure 3C and Supplementary Figure S5D). These data supported that PDS-dependent reprogramming of expression profiles was driven by IRF1–7 family of transcription factors, which are involved in cytoplasmic nucleic acid sensing and *IFNB* gene activation (34).

**Figure 4.**
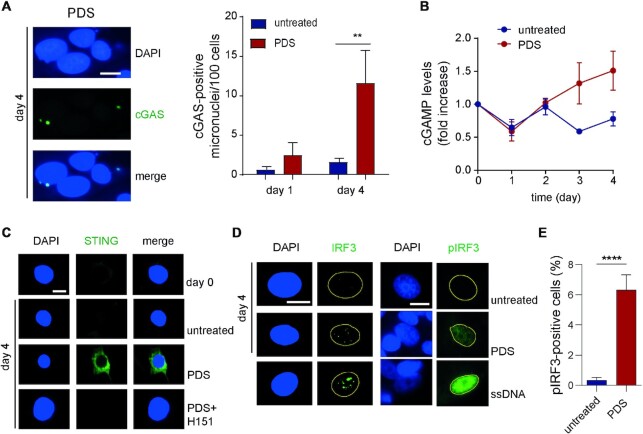
PDS triggers the activation of the cGAS–STING signaling pathway. (**A**) IF images of cGAS-bound micronuclei in MCF-7 cells treated with 10 μM PDS (left) and cGAS-positive micronuclei levels in untreated and PDS-treated cells (right). Data show means of three biological replicates. Significance was determined with a multiple paired *t*-test: **P-value <0.01. (**B**) Levels of cGAMP dinucleotide over time in untreated and PDS-treated MCF-7 cancer cells. cGAMP levels were determined using the Direct 2′,3′-Cyclic GAMP Enzyme Immunoassay Kit. Data show means ± SEM of at least three biological replicates. The *P*-value (determined with the Mann–Whitney test) is 0.057 at day 4. (**C**) IF image of STING in MCF-7 cancer cells as indicated [H151 (2μM) is a STING inhibitor]. (**D**) IF images of IRF3 and pIRF3 (phosphorylated Ser396 IRF3) in MCF-7 cancer cells as indicated. ssDNA indicates cells transfected with salmon sperm DNA as a positive control. (**E**) Fraction of pIRF3-positive (>2 nuclear foci) MCF-7 cells at day 4. Data show means ± SEM of two biological replicates. Significance was calculated by the chi-square test: ^****^P-value <0.0001. Scale bar is 10 μm.

Thus, we next asked whether PDS-induced micronuclei were a source for cytoplasmic DNA activating the cGAS/STING/IRF3 pathway (37). Immunofluorescence microscopy (IF) showed that cGAS was bound to 25–40% of micronuclei induced by PDS, and that cGAS-positive micronuclei increase over time (Figure 4A). Moreover, cellular levels of cGAMP, a signaling molecule produced by activated cGAS (38), also increased in PDS-treated cells (Figure 4B). Thus, the data show that PDS-induced micronuclei are recognized by cGAS, which becomes activated catalyzing the synthesis of cGAMP (38). As cGAMP can signal the presence of cytoplasmic DNA to STING, we then wondered whether STING is also activated. IF images showed that STING signal was increased showing a perinuclear asymmetric pattern in PDS-treated cells as compared to untreated cells at day 4 (Figure 4C) and day 1 (Supplementary Figure S6A), suggesting a translocation of STING to the Golgi apparatus (39). In addition, a specific inhibitor of STING, H151 [which can block STING palmitoylation needed for its activation (40)], could substantially abolish STING localization to the Golgi (Figure 4C and Supplementary Figure S6A). As STING activity leads to the activation of IRF3 through phosphorylation (41), we then determined IRF3 status in PDS-treated MCF-7 cells. The results showed that IRF3 was translocated to the nucleus in an S396-phosphorylated IRF3 (pIRF3) form (Figure 4D and E and Supplementary Figure S7A). By western blot analyses, we could also detect a slight pIRF3 increase as compared to IRF3 in nuclear protein extracts of PDS-treated cells (Supplementary Figure S7B). Overall, our findings show that non-cytotoxic PDS concentrations can trigger the activation of the cGAS/STING/IRF3 pathway in human MCF-7 cells.

### PDS induction of ISG expression is dependent on STING activation

**Figure 5.**
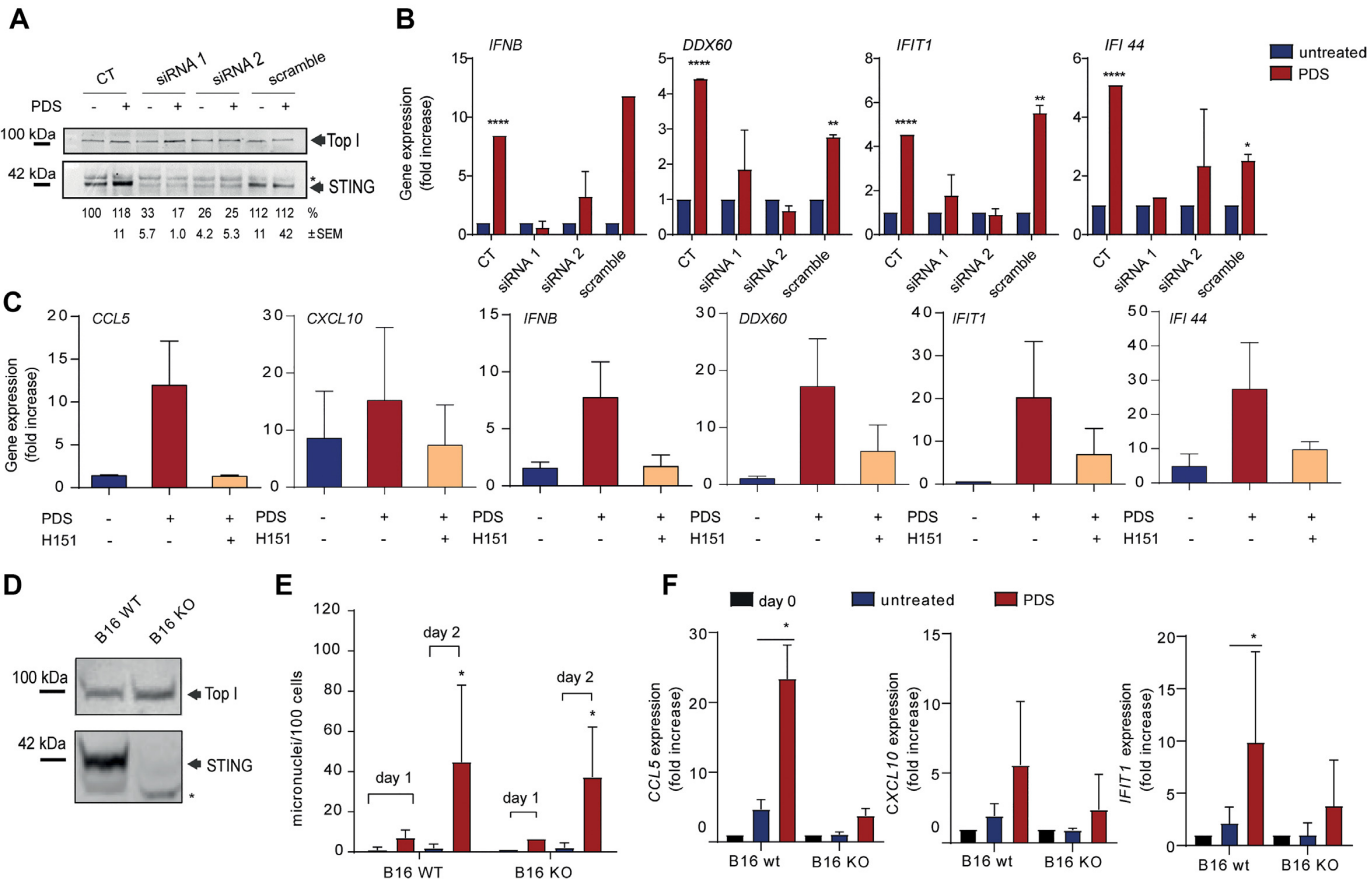
PDS-induced immune gene expression is markedly dependent on STING. (**A**) Western blot analysis showing STING silencing by two different siRNAs after 72 h in MCF-7 cells. Top I levels indicate protein loading. Asterisk indicates non-specific bands. STING band intensity levels from three biological replicates are reported as normalized percentage of untreated samples (untreated control (CT) = 100%; normalized on Top I intensity). Statistical significance was determined with a *t*-test: *P*-value <0.05 and <0.001 for untreated and PDS samples, respectively, for siRNA1; *P*-value <0.01 for untreated and PDS samples of siRNA 2. (**B**) Gene expression of the indicated immune genes induced by PDS (10 μM) at day 4. Expression levels were determined by RT-qPCR with and without STING silencing. Data show means ± SEM of two biological replicates. (**C**) Immune gene expression levels were determined at day 4 after treatment of MCF-7 cells with PDS (10 μM) or with PDS and H151 STING inhibitor (2 μM). Data show means ± SEM of two biological replicates. (**D**) Western blot analyses of STING content in murine melanoma B16 cells. Western blot analyses were performed in triplicate and STING band intensity quantification showed a 100% reduction in STING-KO B16 cells (*P*-value <0.0001). (**E**) PDS-stimulated micronuclei in wt and B16 cells at days 1 and 2 following PDS 10 μM treatment. Data show means ± SEM of at least three biological replicates. (**F**) Gene expression levels as detected by RT-qPCR of *CCL5*, *CXCL10* and *IFIT1* genes in wt and STING-KO B16 cells treated by PDS (10 μM). Data show means ± SEM of at least three biological replicates. In all graphs, significance was determined with a multiple unpaired *t*-test: *P-value <0.05; **P-value <0.01; ^****^P-value <0.0001.

Then, to demonstrate that the STING pathway is responsible for the induction of *IFNB* and ISG expression by PDS, we determined the effect on gene activation by STING inhibition with siRNAs or H151 inhibitor. Two different specific siRNAs were used and both of them were effective in reducing STING protein levels (Figure 5A). *IFNB* gene and ISG activation were effectively reduced by both siRNAs and H151 in PDS-treated cells (Figure 5B and C), showing that the STING pathway is the main mechanism of the activation of *IFNB* gene and ISG by non-cytotoxic PDS doses in MCF-7 cancer cells.

We then extended the study to murine B16 melanoma cells by using a STING-KO cell line and corresponding wt B16 cells (20). A 24-h treatment with PDS led to a similar increase of micronuclei in both wt and KO B16 cells (Figure 5D and E). PDS also activated STING translocation to a perinuclear region in wt B16 cells, but not in KO cells or wt cells co-treated with H151 (Supplementary Figure S6B and C), showing that PDS can activate STING in a different cell type. Interestingly, the expression of three innate immune genes (*CCL5*, *CXCL10* and *IFIT1*) was activated by PDS in wt B16 cells, but not in KO B16 cells (Figure 5F). Thus, the findings show that STING is also activated by PDS in murine melanoma B16 cells likely leading to an inflammatory immune gene response.

### G4 binders can induce IFNB release from MCF-7 cells at non-cytotoxic concentrations

**Figure 6.**
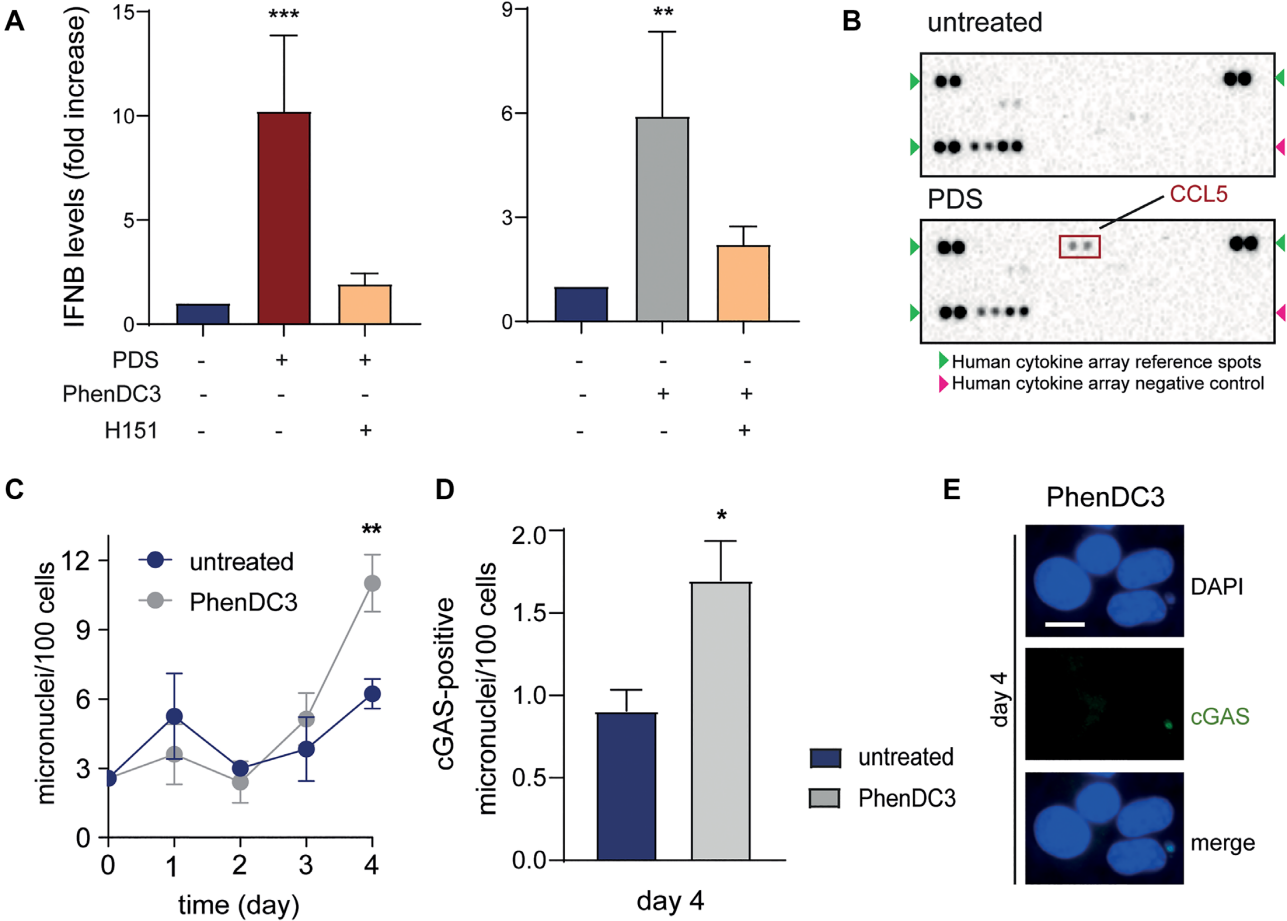
PDS and PhenDC3 induce cytokine secretion mediated by STING activity. (**A**) IFNB levels as detected with an ELISA assay in culture supernatants of MCF-7 cells treated with PDS (10 μM) or PhenDC3 (0.5 μM), with and without co-treatment with H151 STING inhibitor (2 μM). Data show means ± SEM of at least three biological replicates. Significance was determined with the multiple paired *t*-test. (**B**) Levels of cytokines in culture supernatants of MCF-7 cells untreated or treated with PDS (10 μM) at day 4 as detected with a Human Cytokine Array Kit. The red square indicates CCL5 cytokine spots, detected in PDS-treated cell supernatants. A representative immunoblot is shown of two biological replicates. (**C**) Kinetics of micronuclei induction by PhenDC3 (0.5 μM) in MCF-7 cells. Data show means ± SEM of at least three biological replicates. Significance was determined with the unpaired multiple *t*-test. (**D**) cGAS-positive micronuclei induced by PhenDC3 (0.5 μM) in MCF-7 cells at day 4. Data show means of three biological replicates. Significance was determined with the paired multiple *t*-test. (**E**) IF images of cGAS-positive micronuclei induced by PhenDC3 (0.5 μM) at day 4. Scale bar is 10 μm. In all graphs, *P-value <0.05, **P-value <0.01 and ***P-value <0.001.

We then wondered whether gene activation resulted in increased cytokine protein levels in MCF-7 cells treated with PDS. Thus, we measured IFNB and other cytokines secreted into culture medium at day 4. Using an ELISA assay, IFNB protein was increased around 10-fold by PDS, and co-treatment with the STING inhibitor, H151, markedly reduced IFNB production (Figure 6A). We also determined the presence of several other cytokines in culture medium with a membrane blot assay, showing that CCL5 was increased whereas other detected cytokines were not affected by PDS (Figure 6B and Supplementary Figure S7C). The data thus show that PDS has a specific effect on cytokine secretion into culture supernatants.

Next, we asked the question of whether another G4 binder, structurally unrelated to PDS, PhenDC3 (1,5,42), could also affect innate immune gene expression in MCF-7 cells. The results showed that IFNB protein levels were increased around 6-fold by PhenDC3 at a non-cytotoxic concentration (0.5 μM) (Supplementary Figure S1A). Interestingly, PhenDC3 effects were dependent on STING activity as its inhibition significantly reduced the amount of secreted IFNB (Figure 6A). Moreover, PhenDC3 was able to trigger a micronuclei increase, even if to a lower extent than PDS, within the same time range (Figure 6C). PheDC3-induced micronuclei were recognized by cGAS (Figure 6D and E). The data therefore show that a structurally distinct G4 binder, PhenDC3, activates *IFNB* in human cancer cells by triggering the formation of micronuclei and activating the cytoplasmic STING signaling pathway.

We next wondered whether a G4 binder can induce an innate immune gene response in non-cancer cells. Therefore, we measured the transcriptional activation of *CCL5*, *CXCL10*, *IFNB*, *DDX60*, *IFI44* and *IFIT1* genes in normal human MRC5 cells as well as IFNB protein levels secreted into cell culture medium. The results show that only *IFNB* gene is activated by PDS in MRC5 cells and that secreted IFNB protein is much less in MRC5 than in MCF-7 cells (Supplementary Figure S8), documenting that cancer cells show a higher immune gene response to PDS than normal MRC5 cells.

### PDS gene signature can predict immunological hot features in human breast tumors

**Figure 7.**
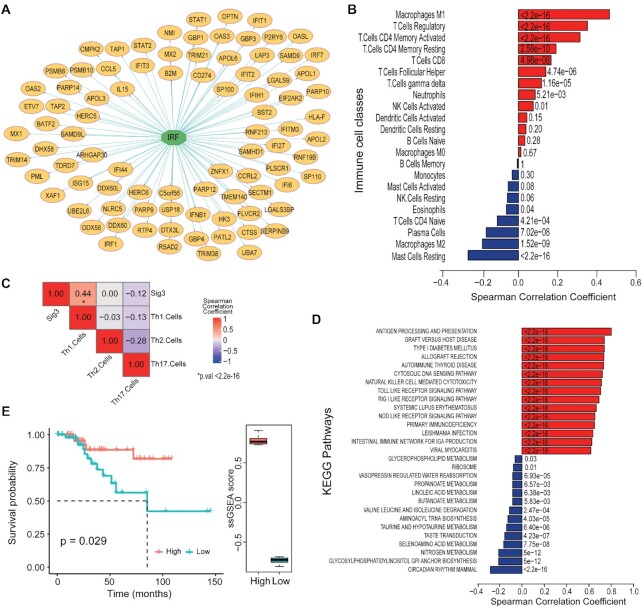
A PDS gene signature can distinguish immunological hot from cold human breast cancers. (**A**) Gene network of Signature-3 genes regulated by IRF family transcription factor. (**B**) Bar plots of Spearman correlation coefficient between ssGSEA Signature-3 and infiltrating immune cells for TCGA BRCA tumor samples (45). *P*-values are indicated for each bar. (**C**) Heatmap showing Spearman correlation between ssGSEA Signature-3 and infiltrating Th1, Th2 and Th17 cells for TCGA BRCA tumor samples. Correlation values are indicated for each comparison. (**D**) Bar plots of Spearman correlation coefficient between ssGSEA Signature-3 and ssGSEA KEGG pathway scores for TCGA BRCA tumor samples. Only top 15 and bottom 15 pathways are shown. *P*-values are indicated for each bar. (**E**) Survival plot of progression-free interval of TCGA BRCA tumor samples grouped into two classes with high (red line and box plot, 95th percentile) or low (blue line and box plot, 95th percentile) ssGSEA enrichment score for Signature-3. *P*-values of the log-rank test are shown in the plot.

As STING activation can exert either immune antitumor-suppressive or -stimulating effects depending on context and duration (43,44), we wondered whether STING-mediated transcriptional profiles induced by PDS were associated with immunological hot or cold features in human breast cancers. Therefore, we analyzed gene expression data of 1064 primary breast tumor samples from the GDC TCGA project (45). Using the PDS upregulated gene (n=727) list, cluster analysis of co-expression of these genes across BRCA tumor samples enabled us to identify three distinct subsets of PDS upregulated genes based on expression patterns in TCGA BRCA samples (Supplementary Figure S9A and Supplementary Data SD11). Among them, the master regulation prediction showed that one subset, Signature-3 set (102 genes), was mostly composed of genes regulated by the IRF transcription factor family, whereas Signature-2 (236 genes) and Signature-1 (266 genes) sets were not enriched for gene targets of one or few transcription factors (Figure 7A and Supplementary Data SD12–SD14). Interestingly, correlation analyses of Signature-3 set enrichment score and tumor immune features in BRCA samples showed a strong correlation of Signature-3 score with infiltrating macrophage M1, T-cell regulatory and follicular helper T cells (Figure 7B-C). These immune cells were reported to elicit antitumor response (30,46–48). Moreover, Signature-3 score was positively correlated specifically with Th1 (but not Th2 and Th17) CD4 T-cell subpopulations, which has been associated with antitumor response and better prognosis (30,46–48). Signature-1 set showed a much lower association with immune cell infiltration and pathway upregulation (Supplementary Figure S9B–D), while Signature-2 set showed a very low correlation with most of immune cell types except for a positive correlation with mast cells and Th17 lymphocytes (Supplementary Figure S9B–D), which do not support antitumor effects (46,49). Not only Signature-3 score was strongly associated with infiltrating antitumor immune cells, but it also correlated with upregulated KEGG pathways primarily related to immune responses, antigen presentation and cytosolic DNA sensing pathway (Figure 7D). Signature-3 score showed instead a negative correlation with macrophage M2 and mast cell infiltration (Figure 7B), which were associated with tumor progression and metastasis (47,50). Consistently, a high Signature-3 score could predict a better survival of BRCA tumor patients, whereas a low score predicted a worst outcome (Figure 7E). Signature-1 and Signature-2 did not show a significant prediction value (Supplementary Figure S9E). Interestingly, breast tumor samples with somatic damaging (nonsense, frameshift or missense) mutations in *BRCA1* or *BRCA2* genes showed a higher expression of Signature-3 score than tumors with *BRCA1/2* wt genes (Supplementary Table S3). However, sample numbers are very low for *BRCA* gene mutations (Supplementary Table S3); therefore, these observations need to be confirmed by further studies.

These findings show that the expression of Signature-3 genes is strongly associated with features of immunological hot tumors (30) and with a favorable prognosis in the TCGA breast cancer dataset, suggesting that PDS-dependent STING activation may elicit an antitumor immune response.

## DISCUSSION

Our results demonstrate that both PDS and PhenDC3, established G4 binders, increase micronuclei formation and innate immune genes in cancer cells at cytostatic concentrations. The data provide clear evidence that a major mechanism of the innate immune gene response is the activation of the cytoplasmic cGAS–STING–IRF3 signaling pathway by ligand-induced micronuclei. Moreover, we have established a PDS gene signature from PDS-upregulated genes in human MCF-7 breast cancer cells that can split TCGA BRCA tumors into distinct classes with immunological hot or cold features and diverse survival outcomes. Our findings therefore establish a new mode of action of G4 binders that can have a marked impact on the development of new anticancer therapeutic strategies.

In past decades, the search of new anticancer G4 binders aimed at the discovery of more cytotoxic ligands interfering with specific G4 structures at oncogenes or telomeres (1,5,8). However, G4 binders are often less cytotoxic (IC_50_ values in the μM range for 24-h treatments) in proliferating cancer cells than other DNA-targeting, clinically effective anticancer agents such as alkylating drugs and topoisomerase poisons (IC_50_ values in the nM range for 24h treatments). G4 binders are also known to promote DNA damage, which can be associated with autophagy with contrasting effects on cell killing potency of G4 binders (51–54). Consistently, autophagy-related GO processes are upregulated by PDS in MCF-7 cells (Figure 2B) likely to help removing micronuclei from the cytoplasm (22). A main result of the present study is the discovery of a marked activation of immune gene expression by two G4 binders at non-cytotoxic concentrations. In agreement with previous reports (11,12), PDS can induce DNA damage and trigger micronuclei increases in surviving breast cancer MCF-7 cells (Figure 1B) and osteosarcoma U2OS cells (12). On the other hand, PhenDC3 is reported to be less effective in inducing DNA damage than PDS (1,5,42), while it is able to induce an increase of micronuclei at several hours after cell treatment (Figure 6C–E). Recent data show that PDS and PhenDC3 can have different mechanisms of cytotoxicity as DNA topoisomerase II may mediate DNA damage and cell killing triggered by PDS but not by PhenDC3 (55). Thus, even though mechanisms of micronuclei formation remain to be defined, our findings establish that micronuclei are induced by non-cytotoxic concentrations of both the binders leading to STING-mediated IFNB production and an innate immune gene response in cancer cells. In contrast, *IFNB* gene was activated by PDS at much lower levels in normal human MRC5 cells (Supplementary Figure S8), indicating that immune gene activation may be specific for tumor cells. Interestingly, in normal neurons, G4 binders have been shown to accelerate cellular aging (56–59) under conditions of persistent *PARP1* activation (56) or prolonged exposure to G4 binders (57). However, we treated cancer cells only once for 24 h and then determined gene expression profiles after 3 days of recovery in drug-free medium. Under these conditions, we have detected a downregulation of the SASP pathway by PDS in MCF-7 cells (Supplementary Figure S5C). Thus, G4 binders can have different molecular and cellular effects in normal versus cancer cells that need to be investigated further and should be considered in drug discovery programs.

Our results also show that ligand-induced activation of innate immune genes and increase of secreted IFNB levels were much reduced, but not fully abolished, following STING inhibition or depletion (Figures 5 and 6). These observations suggest that STING-independent mechanisms may contribute to immune gene response to G4 binders in cancer cells. Similarly to other G4 binders, PDS and PhenDC3 can target different sets of DNA and RNA G4s in living cells resulting in multiple biological effects, including replication interference, transcription and translation impairments, and chromatin changes (1,2,5,8,13). Thus, as ligand effects at cellular levels are likely the result of a balanced interplay of several molecular activities, the target specificity of immunomodulation activity needs to be established in future works on G4-targeting compounds.

Innate immunity (or viral mimicry) is an important factor contributing to the clinical efficacy of standard chemotherapeutic regimen in breast cancer patients (60,61). As different DNA-damaging anticancer agents can stimulate antitumor immune responses, the mechanisms of innate immune activation can, however, be different among different agents (44). As innate immunity activation can prime anti-checkpoint immunotherapy (43), cytostatic G4 binders may stimulate antitumor immunity in unresponsive cold tumors without affecting normal cell vitality. However, as micronuclei can be a source of further genome instability (62,63), future studies need to define fully the mechanisms of genome alterations by micronuclei in cancer cells and its interplay with stimulated antitumor immunity. In addition, as STING and cGAS expression levels can vary substantially among human tumor types (64,65) and the human *STING* gene has allelic haplotypes encoding for protein variants with reduced activity (66,67), G4 binder immunomodulation potency can likely vary among different cancer types and patients. Interestingly, MCF-7 breast cancer cells have a *BRCA1* gene loss (68) that may have contributed to the detected levels of immune gene activation and micronuclei formation. The observation that the PDS gene Signature-3 score is higher in TCGA breast tumors with somatic damaging mutations of *BRCA1* gene (Supplementary Table S3) might suggest that deficiency in homologous recombination pathway may lead to high micronuclei accumulation as observed previously in osteosarcoma U2OS cancer cells (12). The data are in agreement with the BRCAness status of tumors leading to increased genomic instability (69) and, possibly, to innate immune activation as shown previously in breast cancer samples (70). However, further investigations are needed to define the mechanistic of *BRCA1/2* gene status on G4 binder immune gene activation in cancer cells.

In conclusion, the present findings demonstrate that cytostatic doses of two established G4 binders activate a cytoplasmic cGAS- and STING-dependent signaling pathway leading to innate immune gene activation in cancer cells. We propose that cytostatic G4 binders can have a promising immunomodulation activity that may be exploited to increase the therapeutic index of chemo-immunotherapy combinations in cancer patients. Thus, our findings open to the discovery of effective immune-modulating G4 binders and the development of novel chemo-immunotherapeutic anticancer regimen.

## DATA AVAILABILITY

High-throughput sequencing data files can be found at Gene Expression Omnibus GSE161418. Additional resources and data related to this paper may be obtained from authors upon request.

## Supplementary Material

gkab500_Supplemental_Files
