# Supplementary material for: G-quadruplex binders as cytostatic modulators of innate immune genes in cancer cells
Source: Nucleic Acids Res. 2021 Jun 17;49(12):6673–86. doi: 10.1093/nar/gkab500 (PMC8266585; doi:10.1093/nar/gkab500)
Supplement: gkab500_Supplemental_Files [file gkab500_supplemental_files.zip › Supplementary_Figure_Table2405.pdf]

SUPPLEMENTARY MATERIALS

Title

- Full title
- **Cytostatic G-quadruplex binders activate innate immune genes in cancer cells**

- Short title
- **G-quadruplex binders and innate immune genes**

Authors

Giulia Miglietta †, Marco Russo †, Renée C. Duardo and Giovanni Capranico *

Affiliations

Department of Pharmacy and Biotechnology, Alma Mater Studiorum – University of Bologna, via Selmi 3, 40126 Bologna, Italy

† These authors contributed equally to the paper.

* Corresponding author: giovanni.capranico@unibo.it

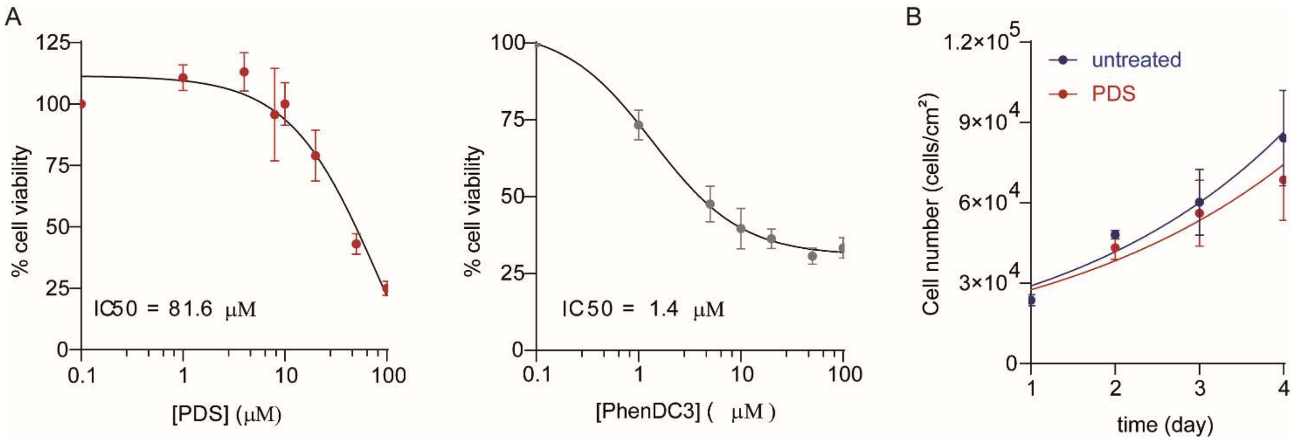

Figure S1. G4 binders (PDS and PhenDC3) cytotoxicity and MCF-7 cells growth curves

A) MTT proliferation assay performed to investigate the PDS and PhenDC3 cytotoxic activity in MCF-7 cancer cells. The graph is reported as the means ± SEM of three independent biological replicates. PDS IC50 is 81.6 μM and PhenDC3 IC50 is 1.4 μM. The results demonstrate that the concentration of 10 and 0.5 μM of PDS and PhenDC3, respectively, used to determine micronuclei formation and IFNB production are non-cytotoxic concentrations; **B)** The graph shows cell growth curves of untreated- and PDS- (10 μM) treated MCF-7 cells. Data are the means ± SEM of three biological replicates. Data showed that PDS at 10 μM does not significantly influence the MCF-7 proliferation rate (cell doubling times are 40 and 46 hours without and with PDS).

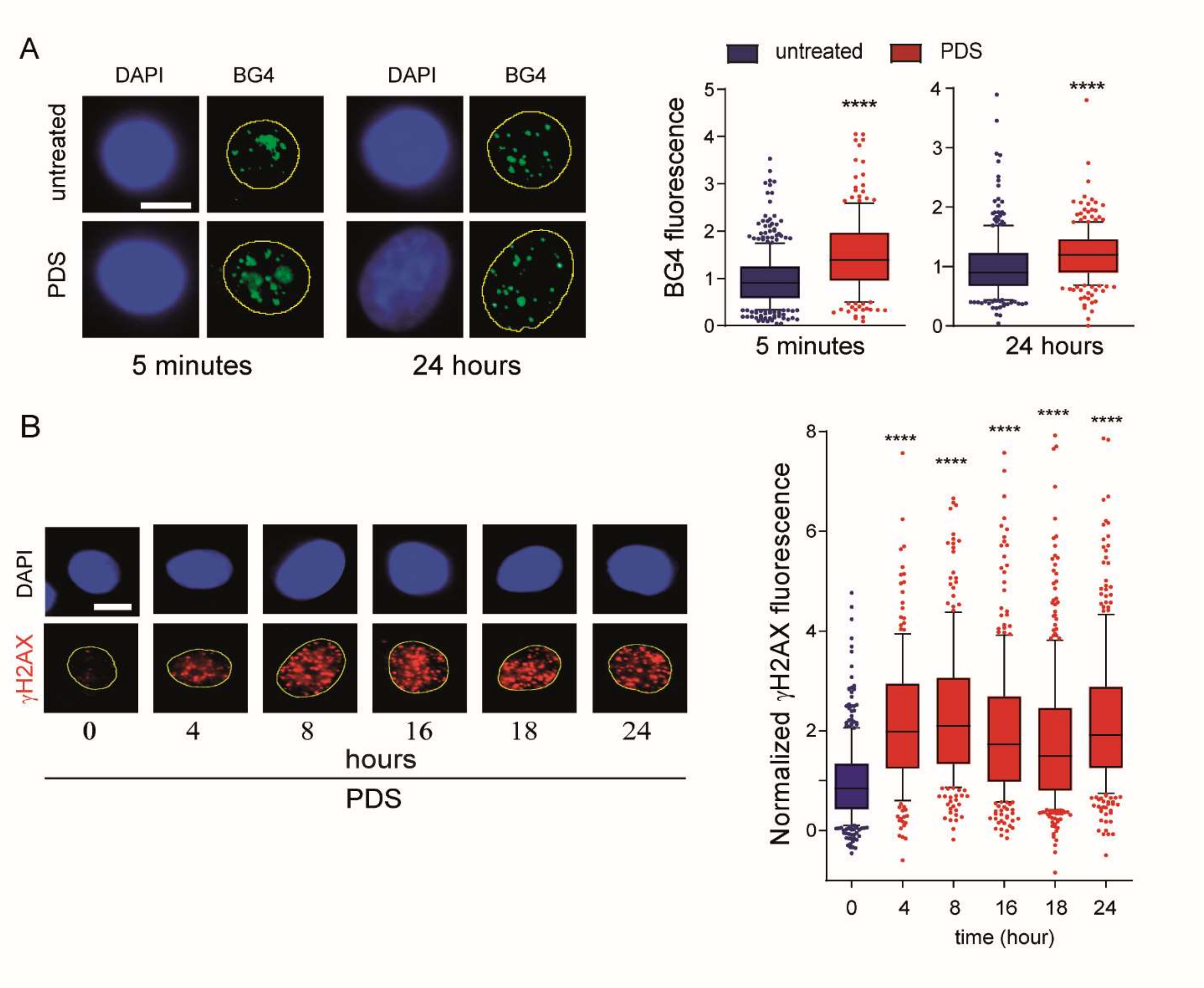

Figure S2. PDS induces G4s and DNA damage in MCF-7 cancer cells

A) Representative images of BG4 immunofluorescence in MCF-7 cells after 5 minutes and 24 hours of PDS (10 µM) (left) and BG4 fluorescence quantification (right). These data showed that PDS promotes G4s formation detected by the specific BG4 scFv antibody after 5 minutes and 24 hours of treatment; **B**) Representative images of DNA damage induction kinetic (detected as γH2AX signal) by PDS (10 µM) after 4, 8, 16, 18 and 24 hours of treatment. The graph represents the γH2AX nuclear fluorescence quantification (right). Immunofluorescence assays clearly demonstrated that PDS results to DSBs in MCF-7 cancer cells after 4, 8, 16, 18 and 24 hours of treatment. Scale bar is 10 µm. The graphs reported are the median of three biological replicates and significance has been calculated by the Kolmogorov–Smirnov parametric test. * p-value<0.05, ** p-value<0.01, *** p-value<0.001, **** p-value<0.0001. The graphical representation of fluorescence quantification is reported as box- (25-75 percentile range) and whisker- (10-90 percentile range) plots in which the horizontal bars represent the median value.

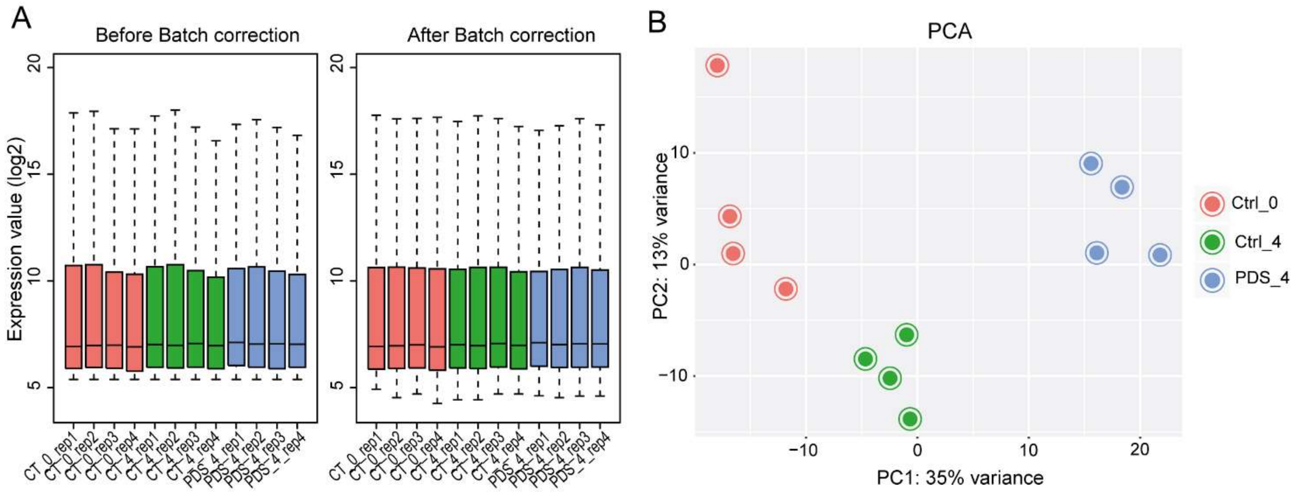

Figure S3. Quality checks of batch-corrected and normalized RNA-seq data

A) Boxplots showing distribution of gene expression values in all sample replicates before and after batch correction step. Batch correction was performed on gene-level counts using *batchcorr* function from limma R package; **B)** PCA plot of RNA-seq read counts of all sample replicates. Replicates are colored by sample as in legend.

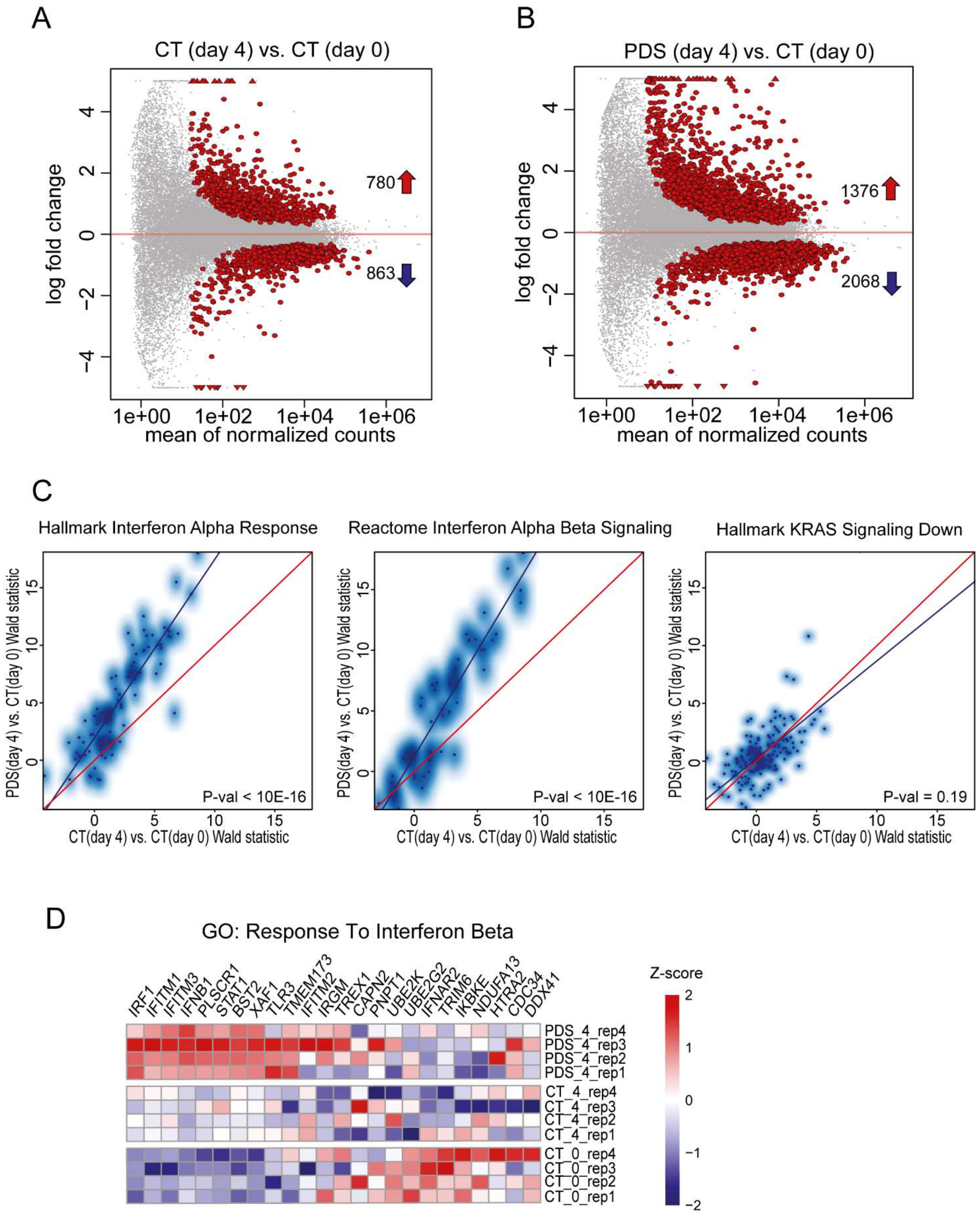

Figure S4. Immune-related pathways are activated somewhat in untreated cells, but prominently in PDS-treated cells

A) MAplot of differentially expressed genes (red dots) in CT (day 4) vs. CT (day 0) contrast (q-value < 0.05). Upward and downward arrows indicate the number of upregulated and downregulated genes, respectively; **B)** MAplot of differentially expressed genes (red dots) in PDS (day 4) vs. CT (day 0)

contrast (q-value < 0.05). Upward and downward arrows indicate the number of upregulated and downregulated genes, respectively. **C)** Scatterplots of Wald-statistic (computed by DESeq2) in CT (day 4) vs. CT (day 0) contrast (x-axis) and PDS (day 4) vs. CT (day 0) contrast (y-axis). Each dot represents a gene belonging to the gene set in plot title. Blue line in each plot represents the regression line of the data. Black line represents the condition in which both contrasts show the same Wald statistic. Statistical significance of the difference between blue and black lines was tested using Kolmogorov-Smirnov test. P-value of this test is reported in each plot; **D)** Heatmap showing Z-scores (column) for each sample (row) of genes belonging to "GO: Response to Interferon Beta" gene set.

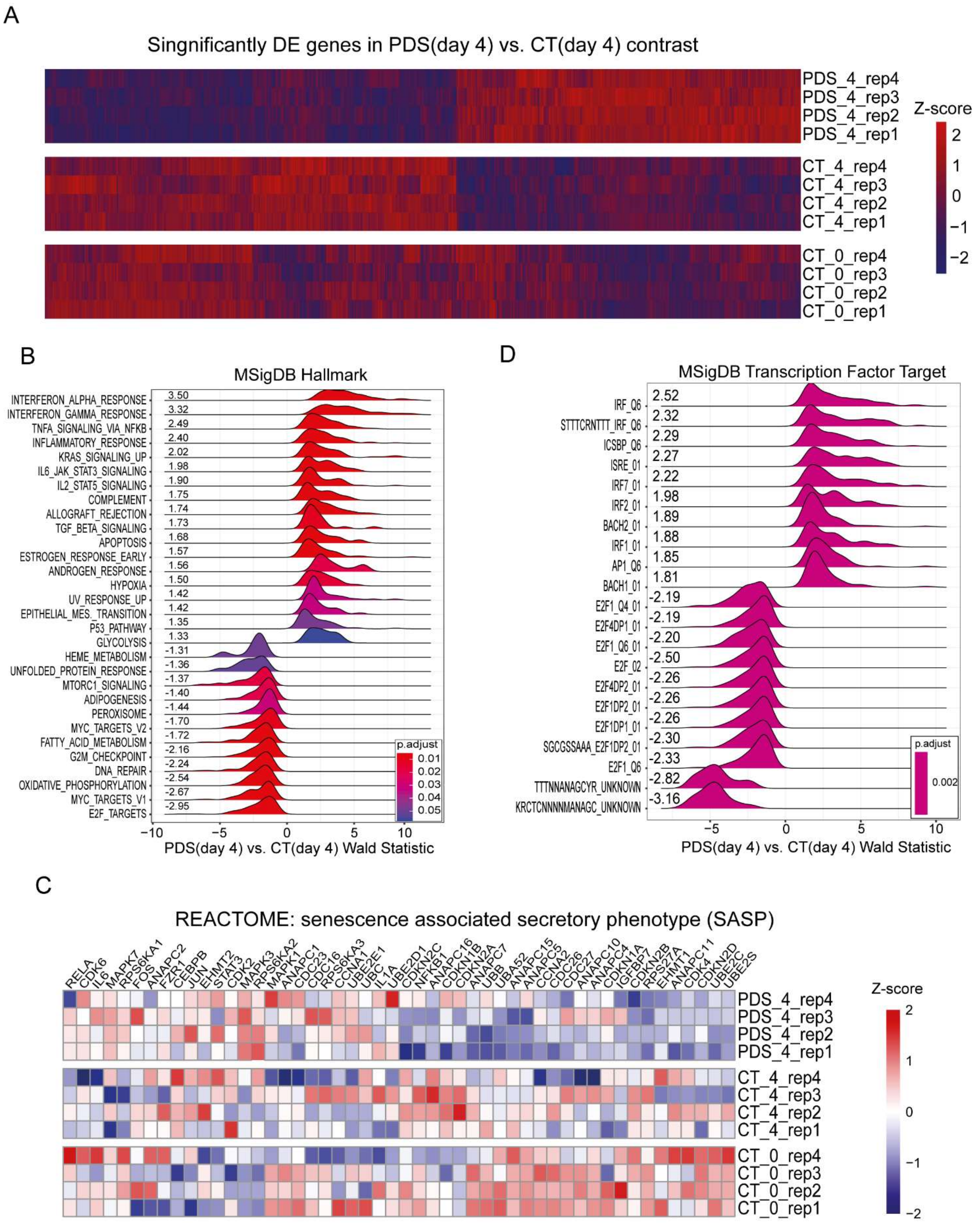

Figure S5. PDS (day 4) vs. CT (day 4) differentially expressed genes and enriched MsigDB genesets

A) Heatmap showing PDS (day 4) vs. CT (day 4) differentially expressed genes Z-scores (rows) for each sample (columns); **B)** Ridgeline plot showing Gene Set Enrichment Analysis (GSEA) results for PDS (day 4) vs. CT (day 4) differentially expressed genes in MsigDB Hallmark genesets. For

each gene set, Normalized Enrichment Score is reported. Ridgelines show the Wald statistic (computed by DESeq2) value of enriched genes for each gene set. Fill color indicates the adjusted p-value of enrichment, as in legend; **C)** Heatmap showing Z-scores (column) for each sample (row) of genes belonging to “REACTOME: senescence associated secretory phenotype (SASP)” gene set (Normalized Enrichment Score: -3.23, adjusted p-value: 0.0129). **D)** Ridgeline plot showing Gene Set Enrichment Analysis (GSEA) results for PDS_t4 vs. CT_t4 differentially expressed genes in MsigDB Transcription Factor Target genesets. For each gene set, Normalized Enrichment Score is reported. Ridgelines show the Wald statistic (computed by DESeq2) value of enriched genes for each gene set. Fill color indicates the adjusted p-value of enrichment, as in legend.

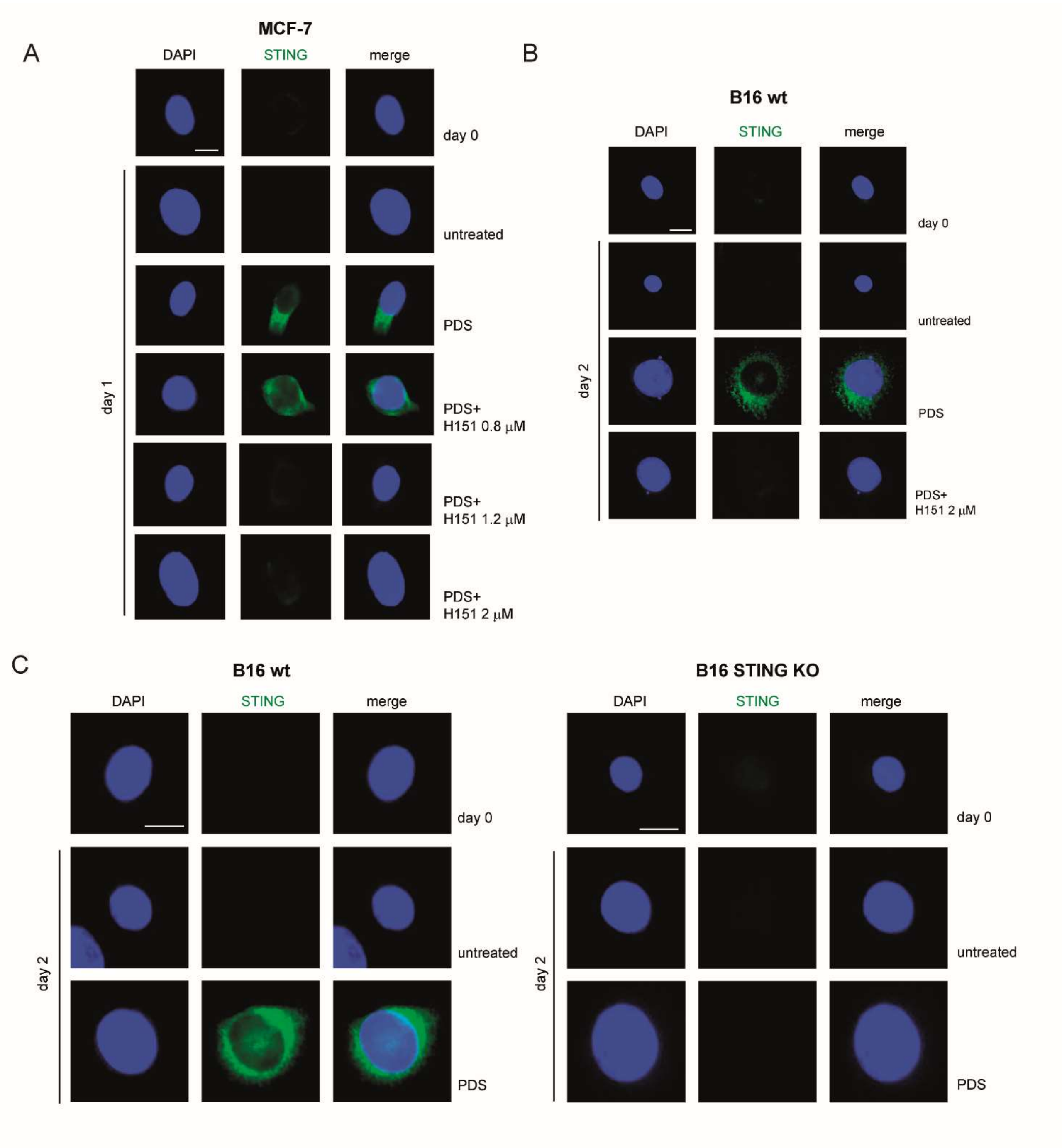

Figure S6. PDS promotes STING activation in MCF-7 and B16 wt and KO cell lines

A) Representative images of STING immunofluorescence assays in MCF-7 cells treated with PDS (10 μM) and co-treated with PDS and H151 STING inhibitor at day 1. The assays showed that PDS promotes an evident perinuclear STING signal that is conceivable with the STING translocation in the Golgi apparatus necessary for its activation. Moreover, the PDS-induced STING signal is partially decreased (PDS+H151 0.8 μM) and fully abolished (PDS + H151 2 μM) by adding the selective STING inhibitor H151 in a dose dependent manner; **B)** Representative images of immunofluorescence assays in the murine melanoma B16 wt cell line at day 2. The STING perinuclear signal is induced by PDS (10 μM) and suppressed by the H151 STING inhibitor (1.2 μM) administration; **C)** STING immunofluorescence assay performed in B16 wt and CRISPR STING

gene knocked out (KO) B16 cell lines. PDS (10 µM) at day 2 increases STING signal only in the B16 wt cell line and not in the KO one. Scale bar is 10 µm.

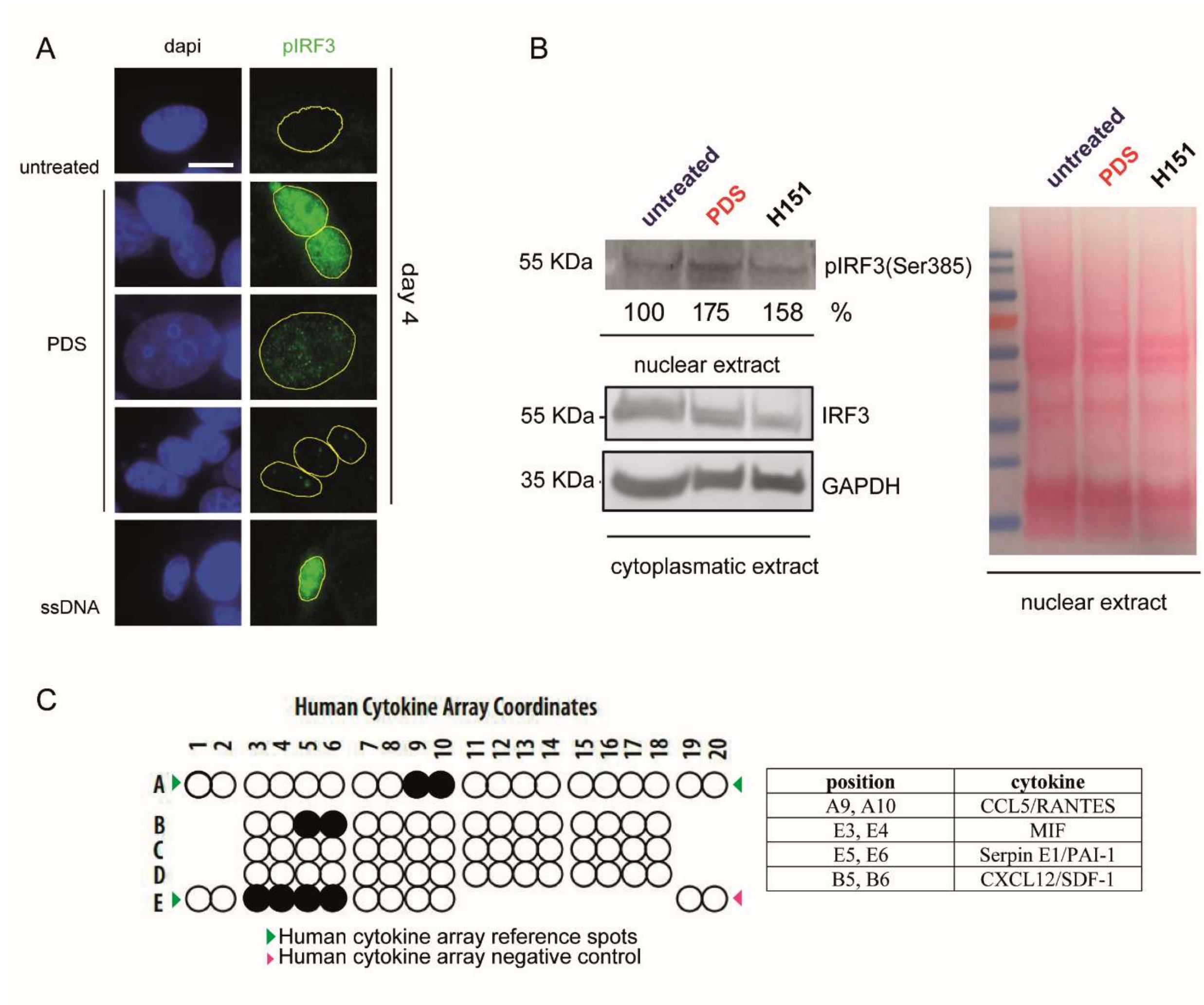

position	cytokine
A9, A10	CCL5/RANTES
E3, E4	MIF
E5, E6	Serpin E1/PAI-1
B5, B6	CXCL12/SDF-1

Figure S7. IRF3 phosphorylation by PDS in MCF-7 and human cytokine array scheme

A) Representative images of pIRF3 immunofluorescence assays in MCF-7 cells treated with PDS (10 μM) or transfected with salmon sperm DNA (ssDNA) for 24 hours. The images represent the variety of patterns in cells treated with PDS or with ssDNA: from a more punctate pattern to a very high fluorescence covering most of the nucleus likely due to the merge of many foci into one large signal (especially in ssDNA). Scale bar is 10 μm. **B)** pIRF3 western blot assay performed in MCF-7 shows that PDS (10 μM) slightly increased the nuclear IRF3 phosphorylation detected by using an antibody that recognized the pIRF3 Ser385 phosphorylation. pIRF3 Ser385 band intensity quantification is reported as percentage (untreated = 100%, normalized on total IRF3). Cytoplasmic extracts have been used to evaluate the level of the not phosphorylated IRF3 form that is not influenced by PDS treatment. As loading control, we reported the Ponceau coloration for the nuclear protein (left) extract and the GAPDH level for the cytoplasmic one; **C)** Human cytokine array legend and positions. The black colored positions are the cytokines detected in both untreated- and PDS- treated arrays (Figure 6B in the main text) and listed in the table (right). Green and pink arrows indicate the array positive (reference spots) and negative controls, respectively.

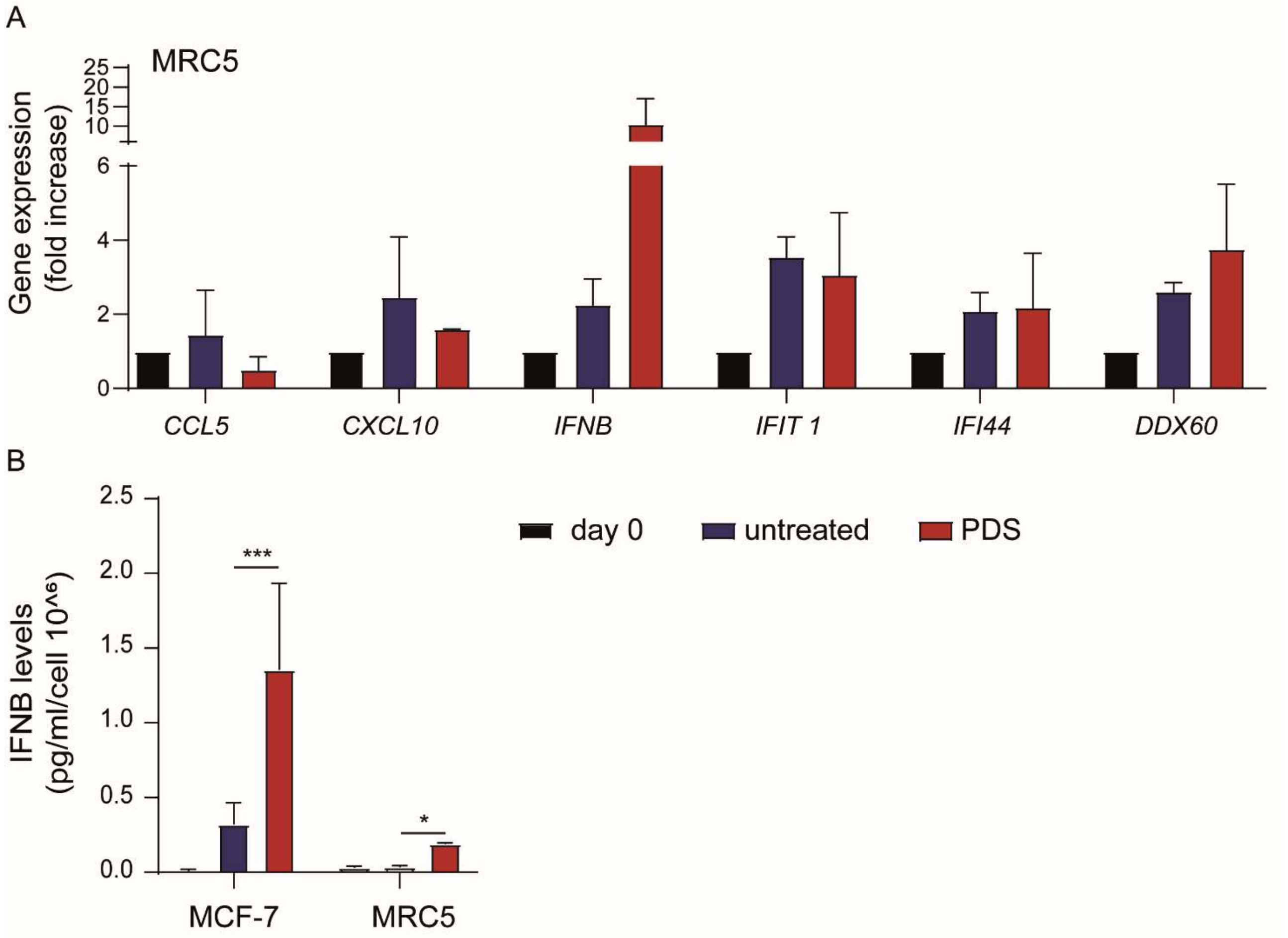

Figure S8. Immune gene expression and IFNB protein levels induced by PDS in normal human MRC5 cell line

A) Gene expression analysis (fold increase) of *CCL5*, *CXCL10*, *IFNB*, *IFIT1*, *IFI44* and *DDX60* in normal human MRC5 cells after PDS treatment (10 µM, at day 3) detected by RT-qPCR. Data are means ±SEM of three biological replicates; **B)** IFNB levels (pg/ml/10^6 cells) detected in the MCF-7 and MRC5 cell culture medium after PDS treatment (10 µM). Data show means ±SEM of three biological replicates and statistical significance was determined with paired *t*-test. *, p-value<0.05; **, p-value< 0.01; ***, p-value<0.001. Increased IFNB protein levels (pg/ml/10^6 cells) are promoted by PDS in normal human MRC5 cells but they are clearly at much lower levels if compared to those detected in MCF-7 cancer cells.

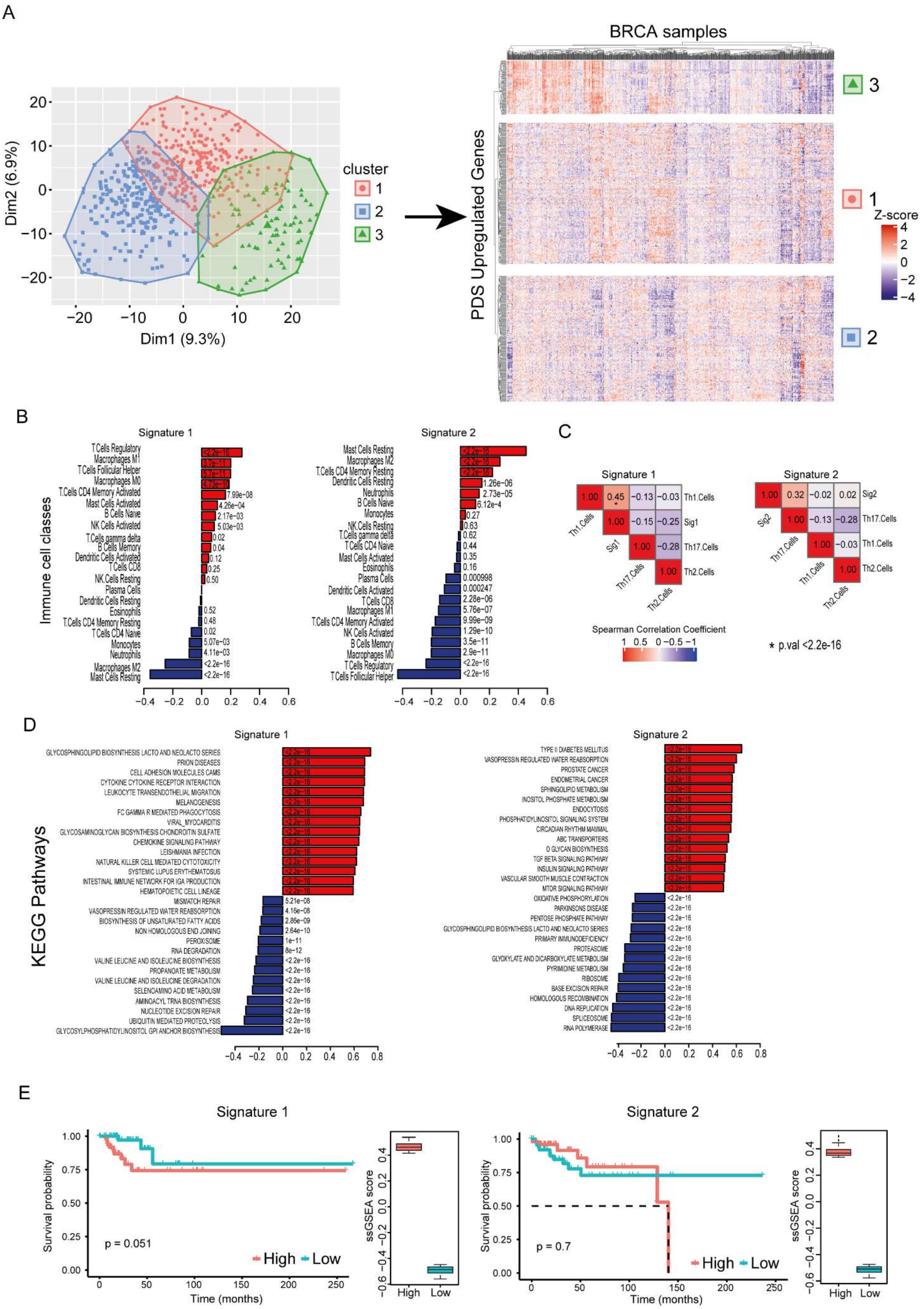
A
BRCA samples
PDS Upregulated Genes
Dim1 (9.3%)
Dim2 (6.9%)
cluster
1
2
3
Z-score
B
Signature 1
Signature 2
Immune cell classes
T.Cells Regulatory
Macrophages M1
T.Cells Follicular Helper
Macrophages M0
T.Cells CD4 Memory Activated
Mast Cells Activated
B.Cells Naive
NK.Cells Activated
T.Cells gamma delta
B.Cells Memory
Dendritic Cells Activated
T.Cells CD8
NK.Cells Resting
Plasma Cells
Dendritic Cells Resting
Eosinophils
T.Cells CD4 Memory Resting
T.Cells CD4 Naive
Monocytes
Neutrophils
Macrophages M2
Mast Cells Resting
7.99e-08
4.26e-04
2.17e-03
5.03e-03
0.02
0.04
0.12
0.25
0.50
0.52
0.48
0.02
5.07e-03
4.11e-03
<2.2e-16
<2.2e-16
Mast Cells Resting
Macrophages M2
T.Cells CD4 Memory Resting
Dendritic Cells Resting
Neutrophils
B.Cells Naive
Monocytes
NK.Cells Resting
T.Cells gamma delta
T.Cells CD4 Naive
Mast Cells Activated
Eosinophils
Plasma Cells
Dendritic Cells Activated
T.Cells CD8
Macrophages M1
T.Cells CD4 Memory Activated
NK.Cells Activated
B.Cells Memory
Macrophages M0
T.Cells Regulatory
T.Cells Follicular Helper
1.26e-06
2.73e-05
6.12e-4
0.27
0.63
0.62
0.44
0.35
0.16
0.000998
0.000247
2.28e-06
5.76e-07
9.99e-09
1.29e-10
3.5e-11
2.9e-11
<2.2e-16
<2.2e-16
C
Signature 1
Signature 2
Th1.Cells
Sig1
Th17.Cells
Th2.Cells
Sig2
Spearman Correlation Coefficient
* p.val <2.2e-16
D
KEGG Pathways
Signature 1
GLYCOSPHINGOLIPID BIOSYNTHESIS LACTO AND NEOLACTO SERIES
PRION DISEASES
CELL ADHESION MOLECULES CAMS
CYTOKINE CYTOKINE RECEPTOR INTERACTION
LEUKOCYTE TRANSENDOTHELIAL MIGRATION
MELANOGENESIS
FC GAMMA R MEDIATED PHAGOCYTOSIS
VIRAL MYOCARDITIS
GLYCOSAMINOGLYCAN BIOSYNTHESIS CHONDROITIN SULFATE
CHEMOKINE SIGNALING PATHWAY
LEISHMANIA INFECTION
NATURAL KILLER CELL MEDIATED CYTOTOXICITY
SYSTEMIC LUPUS ERYTHEMATOSUS
INTESTINAL IMMUNE NETWORK FOR IGA PRODUCTION
HEMATOPOIETIC CELL LINEAGE
MISMATCH REPAIR
VASOPRESSIN REGULATED WATER REABSORPTION
BIOSYNTHESIS OF UNSATURATED FATTY ACIDS
NON HOMOLOGOUS END JOINING
PEROXISOME
RNA DEGRADATION
VALINE LEUCINE AND ISOLEUCINE BIOSYNTHESIS
PROPANOATE METABOLISM
VALINE LEUCINE AND ISOLEUCINE DEGRADATION
SELENOAMINO ACID METABOLISM
AMINOACYL TRNA BIOSYNTHESIS
NUCLEOTIDE EXCISION REPAIR
UBIQUITIN MEDIATED PROTEOLYSIS
GLYCOSYLPHOSPHATIDYLINOSITOL GPI ANCHOR BIOSYNTHESIS
5.21e-08
4.16e-08
2.86e-09
2.64e-10
1e-11
8e-12
Signature 2
TYPE II DIABETES MELLITUS
VASOPRESSIN REGULATED WATER REABSORPTION
PROSTATE CANCER
ENDOMETRIAL CANCER
SPHINGOLIPID METABOLISM
INOSITOL PHOSPHATE METABOLISM
ENDOCYTOSIS
PHOSPHATIDYLINOSITOL SIGNALING SYSTEM
CIRCADIAN RHYTHM MAMMAL
ABC TRANSPORTERS
O GLYCAN BIOSYNTHESIS
TGF BETA SIGNALING PATHWAY
INSULIN SIGNALING PATHWAY
VASCULAR SMOOTH MUSCLE CONTRACTION
MTOR SIGNALING PATHWAY
OXIDATIVE PHOSPHORYLATION
PARKINSONS DISEASE
PENTOSE PHOSPHATE PATHWAY
GLYCOSPHINGOLIPID BIOSYNTHESIS LACTO AND NEOLACTO SERIES
PRIMARY IMMUNODEFICIENCY
PROTEASOME
GLYOXYLATE AND DICARBOXYLATE METABOLISM
PYRIMIDINE METABOLISM
RIBOSOME
BASE EXCISION REPAIR
HOMOLOGOUS RECOMBINATION
DNA REPLICATION
SPLICEOSOME
RNA POLYMERASE
E
Signature 1
Signature 2
Survival probability
Time (months)
p = 0.051
p = 0.7
High
Low
ssGSEA score

Figure S9 Gene expression data exploration of primary breast tumor samples from the GDC TCGA project using PDS upregulated gene signatures

A) Experimental design of gene signatures generation from PDS upregulated genes gene list. K-means clustering (left) of coexpressed genes in TCGA BRCA samples lead to the detection of three distinct gene clusters (right). Expression of PDS gene list were evaluated in all TCGA BRCA tumor samples; **B)** Barplots with Spearman correlation coefficient of ssGSEA Signature-1 and Signature-2 and Immune cells presence in TCGA BRCA tumor samples. Immune cells presence in tumor samples is computed with CIBERSORT. P-values are indicated inside the bars; **C)** Heatmaps showing Spearman correlation of ssGSEA Signature-1 and Signature-2 and Th1, Th2 and Th17 signatures in TCGA BRCA tumor samples. Correlation values are indicated inside each cell; **D)** Barplots with Spearman correlation coefficient of ssGSEA Signature-1 and Signature-2 and ssGSEA KEGG pathway scores in TCGA BRCA tumor samples. Only top 15 and bottom 15 pathways ranked by enrichment score are shown. P-values are indicated inside the bars; **E)** Survival plots of progression free interval (PFI) of TCGA BRCA tumor samples classes that show a high (red line and boxplot, 95th percentile) or a low (blue line and boxplot, 95th percentile) ssGSEA enrichment score for Signature-1 and Signature-2. P-value of log-rank test is reported in the survival plot. Boxplots show the difference between the two groups.

Table S1. Comparison of gene expression increase detected by RT-qPCR and RNA-seq in 6 immune genes in PDS (day 4) vs. CT (day 4) contrast

Gene	RT-qPCR Fold change	RT-qPCR Fold change Standard Error	RT-qPCR Fold change p-value	RNA-seq Fold change (log2)	RNA-seq Fold change Standard Error	RNA-seq Fold change q-value
IFI44	7.93	0.16	0.0321	2.42	0.51	0.000183
CXCL10	7.23	0.10	0.0738	3.81	0.98	0.000118
IFIT1	5.24	0.13	0.0142	2.06	0.42	0.000109
IFNB	5.52	0.05	0.1326	2.16	0.39	0.000005
DDX60	3.94	0.19	0.0172	1.81	0.35	0.000047
CCL5	3.71	0.12	0.0036	1.39	0.28	0.000109

Table S2. Primers used in this work

**Human**		
Gene	Forward	Reverse
IFNB	AGTAGGCGACACTGTTCGTG	GCCTCCCATTCAATTGCCAC
IFIT1	AGGTTCTCCTTGCCCTGAA	AAAGCCCTATCTGGTGATGC
DDX60	CCCAGGGTCCAGGATTTTAT	GAACAGTTGCTGCCACTTGA
IFI44	AGCCTGTGAGGTCCAAGCTA	TTTGCTCAAAAGGCAAATCC
CCL5	CCTCCCCATATTCCTCGGAC	CACACTTGGCGGTTCTTTC
CXCL10	ACTGTACGCTGTACCTGCAT	TGATGGCCTTCGATTCTGGA
CITB	AACTTACTATCCGCCATCCCATAC	AGGGGTTGGCTAGGGTATAATTG
HPRT	TTGCTTTCCTTGGTCAGGCA	ATCCAACACTTCGTGGGGTC
**Mouse**		
Gene	Forward	Reverse
CCL5	TGCTCCAATCTTGCAGTCGT	TCTTCTCTGGGTTGGCACAC
CXCL10	CCAAGTGCTGCCGTCATTTT	AGCTTCCCTATGGCCCTCAT
IFIT1	TGCTCTGCTGAAAACCCAGA	AGGAACTGGACCTGCTCTGA
CITB	ACTGAGAAGCCCCCTCAAAT	ATTCCTTCATGTCGGACGAG

Table S3. Effect of BRCA1 and BRCA2 somatic mutations or copy number loss on Sig_3 enrichment in TCGA BRCA samples

BRCA1 status in TCGA-BRCA samples					
BRCA1 status	N. of samples	mean of Sig_3 NES	SD of Sig_3 NES	median of Sig_3 NES	wilcox.test p.val
Normal	941	0.0006	0.4967	-0.0240	-
Loss	85	-0.0220	0.5201	-0.1427	0.7337
Mutated	20	0.3179	0.4093	0.5301	0.0025
BRCA2 status in TCGA-BRCA samples					
BRCA2 status	N. of samples	mean of Sig_3 NES	SD of Sig_3 NES	median of Sig_3 NES	wilcox.test p.val
Normal	955	0.0047	0.4965	0.0350	-
Loss	77	0.0737	0.5059	0.1352	0.1640
Mutated	14	0.2749	0.5387	0.4319	0.0345

Loss: samples with *BRCA1*/*BRCA2* gene with focal CNV values smaller than -0.3 using Gistic2 software (see https://docs.gdc.cancer.gov/Data/Bioinformatics_Pipelines/CNV_Pipeline/).

Mutated: samples with *BRCA1*/*BRCA2* gene with nonsense, frameshift or missense damaging mutation detected using Mutect2 software (see https://docs.gdc.cancer.gov/Data/Bioinformatics_Pipelines/DNA_Seq_Variant_Calling_Pipeline).
